# PGM3 inhibition shows cooperative effects with erastin inducing pancreatic cancer cell death *via* activation of the unfolded protein response

**DOI:** 10.3389/fonc.2023.1125855

**Published:** 2023-05-16

**Authors:** Barbara Zerbato, Maximilian Gobbi, Tobias Ludwig, Virginia Brancato, Alex Pessina, Luca Brambilla, Andre Wegner, Ferdinando Chiaradonna

**Affiliations:** ^1^ Tumor Biochemistry, Biotechnology and Biosciences, University of Milano Bicocca, Milan, Italy; ^2^ Pathometabolism, Department of Bioinformatics and Biochemistry, Braunschweig Integrated Centre of Systems Biology (BRICS), Technische Universität Braunschweig, Braunschweig, Germany; ^3^ Center for Genomic Science IIT@SEMM, Italian Institute of Technology, Milan, Italy

**Keywords:** hexosamine biosynthetic pathway, unfolded protein response, pancreatic cancer cells, cell death, erastin, ferroptosis

## Abstract

**Background:**

Pancreatic ductal adenocarcinoma (PDAC) is a highly aggressive cancer with a poor patient prognosis. Remarkably, PDAC is one of the most aggressive and deadly tumor types and is notorious for its resistance to all types of treatment. PDAC resistance is frequently associated with a wide metabolic rewiring and in particular of the glycolytic branch named Hexosamine Biosynthetic Pathway (HBP).

**Methods:**

Transcriptional and bioinformatics analysis were performed to obtain information about the effect of the HBP inhibition in two cell models of PDAC. Cell count, western blot, HPLC and metabolomics analyses were used to determine the impact of the combined treatment between an HBP’s Phosphoglucomutase 3 (PGM3) enzyme inhibitor, named FR054, and erastin (ERA), a recognized ferroptosis inducer, on PDAC cell growth and survival.

**Results:**

Here we show that the combined treatment applied to different PDAC cell lines induces a significant decrease in cell proliferation and a concurrent enhancement of cell death. Furthermore, we show that this combined treatment induces Unfolded Protein Response (UPR), NFE2 Like BZIP Transcription Factor 2 (NRF2) activation, a change in cellular redox state, a greater sensitivity to oxidative stress, a major dependence on glutamine metabolism, and finally ferroptosis cell death.

**Conclusion:**

Our study discloses that HBP inhibition enhances, via UPR activation, the ERA effect and therefore might be a novel anticancer mechanism to be exploited as PDAC therapy.

## Introduction

Pancreatic ductal adenocarcinoma (PDAC) is a highly aggressive cancer with a poor patient prognosis (1). Conventional therapy, for both resectable and unresectable PDAC, relies mainly on the use of the chemotherapeutic agent gemcitabine (GEM) either alone or in combination with adjuvant therapies such as paclitaxel conjugated to albumin, 5-FU, capecitabine, and erlotinib, which can increase median OS as compared with GEM monotherapy (2). Despite the fact that drug combinations improve the median survival rate compared to GEM alone, these novel combinations elicit resistance within weeks and hence fail to bring tumor regression. For this reason, there is an urgent need to search for new pharmacological targets that will boost the sensibility to current treatments and reduce drug resistance.

Tumor metabolic rewiring and metabolic adaptation following drug treatments are typical clues of PDAC (3). Indeed, different reports indicate that reprogrammed metabolism closely regulates PDAC development and chemoresistance (4, 5). Among the different metabolic alterations observed in PDAC, the increased flux through the hexosamine biosynthetic pathway (HBP) tightly linked with glucose and glutamine metabolism has been found as a key metabolic change (6, 7). Worthy of note, the final metabolite generated by the HBP is the uridine 5′-diphospho-*N*-acetyl-D-glucosamine (UDP-GlcNAc), the main substrate for O- and N-protein glycosylation. Both post-translational modifications (PTMs) play critical roles in protein folding, stability, activity, macromolecular interactions, function, and nuclear translocation. Therefore, enhancement of HBP flux, responsible for the aberrant protein glycosylation often observed in different types of tumors including PDAC (8, 9), could represent a new target for tumor therapy.

In this regard, we have recently developed and tested in breast and pancreatic cancer cells as well as *in vivo* models a novel compound, FR054. This molecule is an N-acetyl-glucosamine 6-phosphate analogue capable of diminishing the HBP flux by targeting the HBP enzyme PGM3, leading first to a cell proliferation inhibition and then to cell death (10, 11), underlining the fundamental role of this pathway in breast and pancreatic cancer cell proliferation and survival. Importantly, FR054, if combined with GEM (12) or the pan-KRAS inhibitor BI-2852 (13), significantly enhances PDAC cancer cells’ sensitivity to both drugs, also suggesting a role of HBP in drug resistance.

Our previous findings indicated that the death mechanism induced by FR054 is dependent on the acute activation of the unfolded protein response (UPR). Indeed, HBP flux reduction, decreasing UDP-GlcNAc availability, causes a reduction in protein N-glycosylation, an accumulation of misfolded proteins, and finally irremediable cell damage, which drives CHOP-dependent apoptotic signaling (11, 12). Worthy of note, previous reports propose that the UPR is constitutively active in PDAC and it may contribute to the disease progression and the acquisition of resistance to therapy (14). Thus, these different findings highlight a dual role for UPR in correlation with its level of activation. In particular, it is either an adaptive cellular mechanism to weaken protein and metabolic stresses created by a hypoxic environment and to induce chemoresistance, or it is an apoptotic inducer when prolonged in time (15). For this reason, some authors have suggested that targeting UPR in cancer including in PDAC may be highly beneficial, especially in combinatorial treatments that could provide an effective anti-tumorigenic response in patients.

Therefore, in this study, we aimed to further detail the mechanism of FR054-induced cell death, in order to verify whether its pro-apoptotic ability could be enhanced by co-targeting a specific protein or cellular pathway. For this purpose, we performed an RNA-seq analysis in two different PDAC cell lines, namely, MiaPaCa-2 and BxPC3, treated with FR054 to provide unbiased mechanistic insights. Results demonstrate that FR054 treatment induces expression of genes related to UPR, to the NRF2 pathway, and to glutathione biosynthesis and ferroptosis. Consequently, we show that inhibition of glutathione biosynthesis, by using the specific inhibitor for the Solute Carrier Family 7 Member 11/xCT (SLC7A11), ERA, in combination with FR054, significantly enhanced the FR054 effect causing a noteworthy increase in cancer cell proliferation arrest and death. Remarkably, the latter effect was tightly associated with a higher expression of the pro-apoptotic protein DNA Damage Inducible Transcript 3/CHOP (CHOP), reduced activity of SLC7A11 transporter, an alteration of intracellular glutathione (GSH) and glutathione disulfide (GSSG) levels, and a strengthening of lipid peroxidation, indicating that SLC7A11 inhibition is synthetic lethal with FR054. Notably, the metabolic analysis supported the role of FR054 in favoring glutamine metabolism over glycolysis. Therefore, our work reveals that simultaneous inhibition of HBP with FR054 and GSH metabolism could have therapeutic benefits in the treatment of pancreatic cancer.

## Materials and methods

### Materials

N-Acetyl-L-cysteine (NAC), DL-Buthionine-sulfoximine (BSO), and BPTES were purchased from Sigma-Aldrich (Merck Life Science, Milan, Italy), while erastin (ERA) was purchased from Selleckchem (Planegg, Germany). FR054 was synthesized either by our laboratories or by WuXi AppTec Co., Ltd. (Tianjin, China) (10, 11).

### Cell lines

Human pancreatic ductal adenocarcinoma cell lines MiaPaCa-2 and PANC1 were routinely cultured in high glucose Dulbecco’s medium Eagle’s medium (DMEM), while the BxPC3 cell line was cultured in RPMI (Euroclone, Milan, Italy). Both media were supplemented with 2 mM L-glutamine, 100 U/ml penicillin, 100 μg/ml streptomycin (Sigma-Aldrich, Merck Life Science, Milan, Italy), and 10% fetal bovine serum (FBS; Euroclone, Milan, Italy). The cells were grown and maintained according to standard cell culture protocols and kept at 37°C with 5% CO_2_. The medium was replaced every 2–3 days and cells were split or seeded for experiments when they reached the sub-confluence. MiaPaCa-2, PANC1, and BxPC-3 were originally obtained from the American Type Culture Collection (ATCC).

### Trypan blue vital assay

Where not differently specified, for experiments, cells were seeded in the complete growth medium, and after 24 h, cells were washed twice with phosphate buffer saline 1X (PBS 1X, Euroclone, Milan, Italy) and incubated in a complete medium with or without FR054. Twenty-four hours later, cells were also treated with or without 10 µM ERA.

Trypan blue vital assay was performed by seeded 4 × 10^4^ (MiaPaCa-2 and PANC1) and 8 × 10^4^ (BxPC3) viable cells per well in 12-well dishes, respectively. Inhibition of proliferation and cell death were assessed at different time points (48 and 72 h), counting harvested cells with Burker chamber after staining with trypan blue 0.4% (Life Technologies–Thermo Fisher Scientific, Waltham, MA, USA). In particular, cells were incubated in a complete medium with or without 350 µM FR054 for 24 h and then, after further 24 h, were also treated with or without 10 µM BPTES or 10 µM erastin. The MiaPaCa-2 cell line was also tested with two different concentrations of FR054, namely, 350 µM or 500 µM, to define an optimal concentration for all the other experiments in all PDAC cell lines. Cell death was also measured at 72 h of treatments as previously described, using a different treatment scheme: 24 h after seeding, cells were incubated in a complete medium with or without 350 µM FR054, and at 48 h post-seeding, cells were also treated with or without 10 µM ERA and 5 mM NAC or 5 mM BSO.

### Western blot analysis

MiaPaCa-2 (6 × 10^5^ cells) and BxPC3 (1.2 × 10^6^ cells) were seeded onto 100-mm dishes in the complete growth medium. After 48 and 72 h, cells were harvested and disrupted in a buffer containing 50 mM Tris-HCl, pH 7.6, 150 mM NaCl, 1% (v/v) Triton X-100, 0.2% (v/v) sodium dodecyl sulfate (SDS), 0.5% (v/v) sodium deoxycholate, 1 mM MgCl_2_, 1 mM EDTA, protease inhibitor cocktail, and phosphatase inhibitors (Sigma-Aldrich, Milan); 20 µg of total proteins was resolved by SDS-PAGE and transferred to the nitrocellulose membrane, which was incubated overnight with specific primary antibodies: cleaved caspase-3 (#9662S, 1:500), GRP78 (BiP, #3177, 1:1,000), phospho-eIF2α Ser 51 (#3398, 1:1,000) from Cell Signaling Technology Inc. (Euroclone, Milan, Italy), eIF2α (sc-133127, 1:1,000) from Santa Cruz Biotechnology Inc. (DBA Italia, Milan, Italy), ATF4 (#11815, 1:800), CHOP (#2895, 1:1,000) from Cell Signaling Technology Inc. (Euroclone, Milan, Italy), NRF2 (sc-13032, 1:1,000) from Santa Cruz Biotechnology Inc. (DBA Italia, Milan, Italy), SLC7A11 (ab175186, 1:5,000) from Abcam (Cambridge, UK), HO-1 (HMOX1; #86806, 1:500) from Cell Signaling Technology Inc. (Euroclone, Milan, Italy), and Keap1 (sc-365626, 1:1,000) and Vinculin (#sc-5573, 1:10,000) from Santa Cruz Biotechnology Inc. (DBA Italia, Milan, Italy). Levels of protein expression on Western blots were quantified by densitometry analysis using ImageJ software.

### Glutamic acid and cystine determination

Cells were plated at a density of 4 × 10^4^ (MiaPaCa-2 and PANC1) and 8 × 10^4^ (BxPC3) per well in 12-well dishes. The media used for subsequent HPLC analyses were collected after 48 h and 72 h of treatment. Glutamic acid and cystine concentrations were determined by chromatographic analysis using a Jasco HPLC system (Jasco Europe, Cremella, Lecco, Italy) under the control of the ChromNAV 2.0 software. The analyses were carried out using the method described by Henderson et al. with some modifications. The derivatized amino acids were separated using a Waters (Milford, MA, USA) XTerra RP18 Column (4.6 mm × 250 mm i.d., 5 μm particle size) equipped with a precolumn Security Guard (Phenomenex, Macclesfield, UK). The flow rate was set at 1 ml/min with the column oven at 40°C. Twenty microliters of the sample, appropriately diluted, was injected into the system and the chromatogram was monitored at 338 nm. The pre-column derivatizing reagent o-Phtalaldehyde (OPA) was prepared by dissolving 25 mg of OPA and 25 mg of 3-Mercaptopropionic acid in 5 ml of 0.2 M borate buffer, pH 10.2, and stored at 4°C until any sign of degradation. The samples were prepared by adding 5.5 μl of the derivatization reagent to 5.5 μl of the samples and bringing the volume up to 200 μl with water. HPLC separation employed mobile phases A (40 mM sodium phosphate buffer, pH 7.8) and B (30% acetonitrile:60% methanol:10% H_2_O). The column was equilibrated with 94.5%/5.5% (v/v) mobile phase A/B for 10 min before each sample analysis. The elution gradient consisted of 94.5%/5.5% (A/B) for 0.85 min; 2.15-min linear gradient to 13% phase B; 23-min linear gradient to 54% phase B; 94.5%/5.5% (A/B) for 4 min. Amino acid peaks were identified by comparing their retention times with that of the standard mixture. At the end of every set of samples, the column was washed with 20% acetonitrile–80% water for 30 min to remove any trace of salts, and then with 80% acetonitrile–20% water for 20 min and stored in solution.

### Cell morphology

MiaPaCa-2, PANC1, and BxPC3 cells were plated onto 100-mm dishes in the complete growth medium at a density of 6 × 10^5^, 6 × 10^5^, and 1.2 × 10^6^ per well, and they were treated with 350 µM FR054 after 24 h. At 48 h post-seeding, the cells were treated with 10 µM ERA. The images were collected with Olympus CX40 phase contrast microscope using a 20X objective using X_entry Alexasoft Imaging Software.

### GSH and GSSG evaluation

To measure reduced glutathione (GSH) and oxidized glutathione (GSSG) content, MiaPaCa-2, PANC1, and BxPC3 cells were plated in six-well dishes at a density of 1 × 10^5^, 1 × 10^5^, and 2 × 10^5^ per well, respectively. Intracellular GSH and GSSG levels were determined after 48 h of FR054 and erastin in single or combined treatment by VICTOR X3 multimode plate reader (PerkinElmer, Milan, Italy) using a Total GSH/GSSG colorimetric kit (CO-K097-M, Immunological Sciences, Rome, Italy) according to the manufacturer’s instructions. The sample absorbance was measured at 412 nm. Relative GSH and GSSG content was normalized to the cell number.

### BODIPY assay

To evaluate lipid peroxidation, the BODIPY 581/591 C11 dye (Life Technologies–Thermo Fisher Scientific, Waltham, MA, USA) staining was carried out. One day before treatment, MiaPaCa-2, PANC1 (5 × 10^3^ cells) and BxPC3 (1 × 10^4^ cells) were seeded onto a black 96-well plate in the complete growth medium. Then, cells were incubated in the complete medium with or without 350 µM FR054. After 24 h, cells were also treated with or without 10 µM ERA. All cell lines were also treated with 5 mM NAC or 5 mM BSO as controls for the presence of oxidative stress. Lipid peroxidation level was measured after 48 h of treatment. Cells were washed twice with PBS 1X and incubated in PBS 1X with 10 µM BODIPY C11 dye for 15 min at 37°C. Then, cells were washed again with PBS 1X, and the dye fluorescence was measured. Oxidation of BODIPY C11 resulted in a shift of the fluorescence emission peak from 590 nm to 510 nm proportional to lipid ROS generation and was analyzed using FLOUstar^®^ Omega (BMG Labtech, Germany).

### MDA assay

To evaluate the formation of the lipid peroxidation product, malondialdehyde (MDA), the cells were seeded at a density of 6 × 10^5^ (MiaPaCa-2 and PANC1) or 1.2 × 10^6^ (BxPC3) onto 100-mm dishes in the complete growth medium. The MDA levels in the cells were measured after 48 h of FR054 and ERA in single or combined treatment using MDA Colorimetric assay kit (CO-K028-M, Immunological Sciences, Rome, Italy) according to the manufacturer’s instructions. The sample absorbance was measured at 532 nm by VICTOR X3 multimode plate reader (PerkinElmer, Milan, Italy) and the relative MDA concentration was normalized to the protein content.

### Stable isotope labeling and GC/MS

All extraction experiments were performed within 3 weeks upon cell thawing. The cells were plated in six-well dishes at a density of 9 × 10^4^ cells/well for MiaPaCa-2 and PANC1 cell lines or 1.1 × 10^5^ cells/well for the BxPC3 cell line. After 24 h of seeding, the media were replaced with DMEM for mass spectrometry (Sigma-Aldrich, Merck Life Science, Milan, Italy) for MiaPaCa-2 and PANC1 cell lines, and with MS-Grade SILAC RPMI 1640 (Gibco, Thermo Fisher Scientific, Waltham, MA, USA) medium, without Phenol Red, L-Arginine, and L-Lysine, which were manually added at a concentration of 200 mg/ml and 40 mg/ml, respectively, for the BxPC3 cell line. In both media, 10% sterile-filtered dialyzed FBS was added (Gibco, Thermo Fisher Scientific, Waltham, MA, USA). For stable isotope labeling, in media, U13C Glucose and unlabeled Glutamine or U13C Glutamine and unlabeled Glucose (Sigma-Aldrich, Merck Life Science, Milan, Italy) were also added. After 48 h of FR054 and ERA in single or combined treatment, the medium of each condition was collected, and wells were washed with 0.9% NaCl solution to perform metabolite extraction following standard laboratory procedure as described in Ref (16).. Then, the polar phase (upper phase) was collected, dissolved in MeOH/IS water, and transferred to a GC/MS glass vial. The eluates were evaporated in a vacuum concentrator system (CentriVap; Labconco, Kansas City, MO, USA) and stored at −20°C until further analysis. Targeted analysis was carried out on an Agilent 7890B Gas Chromatograph equipped with a 30-m DB-35MS + 5-m Duraguard capillary column. Helium was used as carrier gas at a flow rate of 1 ml per minute. The system was connected to an Agilent 5975 Mass Spectrometer (Agilent Technologies, Santa Clara, CA, USA). Derivatization reagent N-tert-butyldimethylsilyl-N-methyltrifluoroacetamide, 1% tert-butyldimethylchlorosilane (MTBSTFA with 1% t-BDMCS; Restek Corporation, Bellefonte, PA, USA), and methoxylamine (MeOX), dissolved in pyridine at a concentration of 20 mg/ml, were added to protect metabolites. Mixing was carried out by an autosampler dissolving in MeOX/pyridine and shaking at 40°C for 90 min. Afterward, derivatization agents were injected into vials, shaken for 30 or 60 min at 40°C, and analyzed. The GC oven was set to 100°C for 2 min, increasing the temperature to 10°C per minute until it reached 300°C. Temperature was maintained for 3 min, raising it then by another 25°C. MS performed the electron ionization at 70 eV, the source had a constant temperature of 230°C, and quadrupoles had a constant temperature of 150°C. Analytes were detected in selected ion monitoring (SIM) mode. Raw GC/MS data were analyzed on MetaboliteDetector, an open-source software running on Linux-based operative systems developed by Karsten Hiller et al. (17). Chemical formulas for mass isotopomer distribution (MID) determination were taken from (18).

### Gene expression analysis

Two biological replicates were used for gene expression analysis. In particular, MiaPaCa-2 and BxPC3 cell lines were seeded at a density of 5 × 10^6^ cells in 100-mm dishes and, after 24 h, were treated with 350 µM FR054 and incubated for 48 h. Then, the cells, untreated and treated, were pelletized and the mRNA was extracted by using Qiagen RNeasy Kit (X) starting from 5 × 10^6^ cells/sample. RNA-seq experiments, data extraction, and analysis were performed in outsourcing (GALSEQ, Milan, Italy). In particular, total RNA was quantified using a spectrophotometer and RNA integrity was evaluated using the Agilent 4200 TapeStation. RNA-stranded libraries were generated starting from 500 ng of total RNA, and mRNA was selected with poly-T oligo attached magnetic beads using an Illumina TruSeq^®^ Stranded mRNA Library Prep kit (Catalog # 20020594). Libraries were quantified and quality checked on an Agilent 4200 TapeStation system (High Sensitivity D1000), and then sequenced on an Illumina NovaSeq 6000 platform with paired-end reads 150 bp long, with a depth of 30 million clusters/sample. Fastq reads were quality checked using FastQC (https://www.bioinformatics.babraham.ac.uk/projects/fastqc/) for overall and per-base read quality and presence of adaptor sequences. Paired reads were subsequently aligned to the human reference genome (GRCh38/hg38) using the splice-aware aligner STAR v 2.5.0c and indexed with Samtools. Per-gene read counts were generated using the STAR quantMode geneCounts option. Raw per-gene read counts were then processed using the Bioconductor package DESeq2 package v. 1.30 (19) in order to perform the differential expression analysis. Genes with an adjusted *p*-value [Benjamini–Hochberg false discovery rate (FDR)] < 0.05 and log2FC <−2 or >2 were considered as differentially expressed. Sorted, indexed bam files were finally used for manual quality check of the alignment profiles. The RNA-seq data have been deposited at the NCB-GEO database, accession number GSE223303.

### Statistical analysis

All statistical analyses were performed using GraphPad Prism v 8.0.2 (GraphPad Software Inc., La Jolla, CA) and data are presented as mean ± SD and as mean ± SEM from three or more independent experiments. For the inhibition of proliferation evaluated after treatment with different concentrations of FR054 and/or ERA/BPTES and for GSH, GSSG, and MDA determination, statistical significance (*p < 0.05, **p < 0.01, ***p < 0.001, ****p < 0.0001) was determined using one-way ANOVA with Tukey’s multiple comparisons test. For GC/MS analysis performed with stable isotope labeling, statistical significance (*p < 0.05, **p < 0.01, ***p < 0.001) was determined with Student’s *t*-test. Instead, for all other experiments, statistical significance (*p < 0.05, **p < 0.01, ***p < 0.001 ****p < 0.0001) was determined using two-way repeated measures ANOVA with Tukey’s multiple comparisons test.

## Results

### Transcriptional analysis reveals upregulation of NRF2 and ferroptosis pathways following PDAC cells treatment with FR054

**Figure 1:**
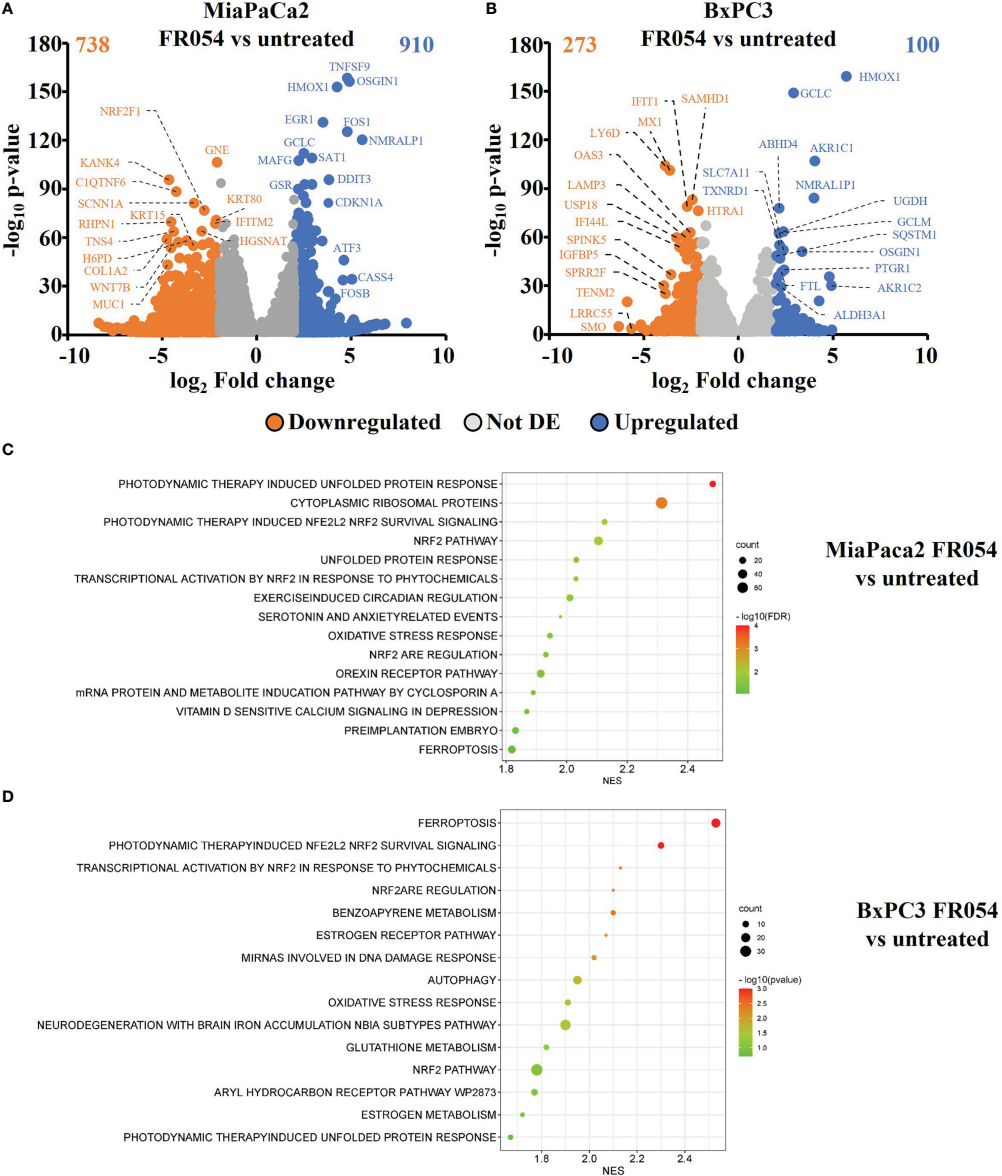
Identification and characterization of DEGs in PDAC cell lines upon FR054 treatment compared to control. **(A, B)** Volcano plot of DEGs identified in MiaPaCa-2- and BxPC3 FR054-treated cells versus untreated cells. Upregulated genes are indicated with cyan dots; downregulated genes are indicated with orange dots; not DEGs are indicated with gray dots. DEGs *p*-value <0.05. The top 15 DEGs are depicted. **(C, D)** Gene set enrichment analysis (GSEA) performed by using the DEGs shown in A and B of MiaPaCa-2- and BxPC3 FR054-treated cells versus untreated cells.

**Figure 2:**
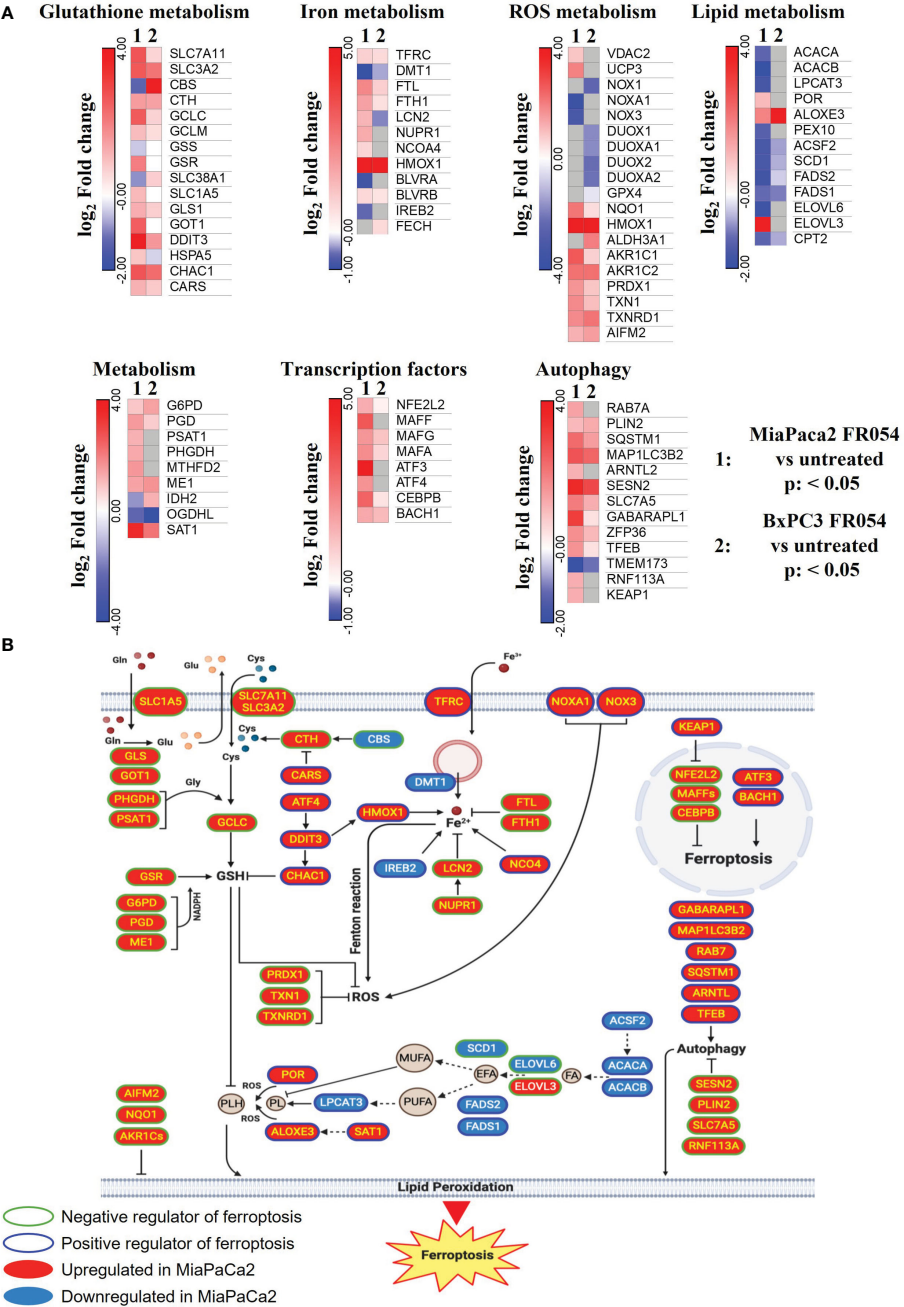
DEGs in MiaPaCa-2- and BxPC3 FR054-treated cells versus untreated cells are enriched of mRNAs involved in oxidative stress response and ferroptosis. **(A)** Dendrograms show genes belonging to the cellular processes described in the figure that are present in the list of the DEGs [*p*-value <0.05, Benjamini–Hochberg false discovery rate (FDR)]. In the heat map, color indicated log_2_ fold change; red represents upregulated genes, blue represents downregulated genes, white denotes no DEGs, and gray indicates missing values. **(B)** The cartoon shows the role of some of the genes identified in **(A)** in the ferroptosis process as described in the main text.

To gain insights into the transcriptional differences between the KRAS PDAC mutated cell line, MiaPaCa-2, and wild-type PDAC cell line, BxPC3, upon FR054 treatment, we compared FR054-regulated transcriptome to untreated samples in MiaPaCa-2 and BxPC3 cells by RNA-seq analyses. To find differentially expressed genes (DEGs), we used mRNAs showing twofold changes with *p*-values smaller than 0.05. The analysis of MiaPaCa-2 cells captured 1,648 DEGs, among which 910 were significantly upregulated and 738 were significantly downregulated in FR054-treated cells as compared to untreated cells (**Figure 1A**, **Supplementary Table 1**). In BxPC3 cells, the analysis identified 373 DEGs, among which 100 were significantly upregulated and 273 significantly downregulated in FR054-treated cells as compared to untreated cells (**Figure 1B**). We found that genes containing antioxidant response element (ARE) regulated by NRF2 protein, including *Heme Oxygenase 1 (HMOX1)*, *Glutamate-Cysteine Ligase Catalytic Subunit (GCLC)*, *Glutamate-Cysteine Ligase Modifier Subunit (GCLM)*, *Spermidine/Spermine N1-acetyltransferase 1* (*SAT1*), *Aldo-Keto Reductase Family 1 Member C1* (*AKR1C1*), *Oxidative Stress Induced Growth Inhibitor 1 (OSGIN1), Glutathione-Disulfide Reductase (GSR), SLC7A11, Thioredoxin Reductase 1 (TXNRD1) Ferritin Light Chain (FTL), Activating Transcription Factor 3 (ATF3), Aldehyde dehydrogenase 3A1 (ALDH3A1)*, and *Alpha/beta-hydrolase domain containing 4 (ABHD4)*, were the top enriched genes in treated cell lines compared with untreated cells. It should also be noted that the other top enriched genes were related to the anti-oxidant response regulated by NRF2 since *MAF BZIP Transcription Factor G* (*MafG*) heterodimerizes with NRF2 and *NmrA Like Redox Sensor 1* (*NMRAL1P1*), which encodes for an NADPH sensor protein, is tightly associated with the NRF2 pathway. In addition, as expected for the well-known capacity of FR054 to induce ER stress and UPR, some transcription factors (TFs), tightly linked to this response, including *DNA Damage Inducible Transcript 3/CHOP (DDIT3)*, *Early Growth Response 1 (EGR1)* and *Activating Transcription Factor 3 (ATF3)*, were more upregulated in MiaPaCa-2-treated cells. To computationally confirm the specific enrichment of some TFs as responsible for the observed changes in gene expression upon FR054 treatment, upregulated DEGs for both cell lines were used as a query to interrogate the ChEA3-ChIP-X Enrichment analysis libraries using the web-based software Enrichr (20). In particular, we used for MiaPaCa-2 cells 700 out of 910 upregulated DEGs with a log_2_FC ≥ 2 and the ReMap library (21). Conversely, since there are few upregulated DEGs for BxPC3 cells (specifically 100, but only 80 were recognized by the database), we decided to also accept for the TF enrichment analysis the upregulated DEGs with a log_2_FC ≥1.5 (upregulated genes used: 137) and the Top rank analysis that gives as readout the integrated results from all the ChEA3-ChIP-X libraries. As shown in **Supplementary Tables 2**, **3**, in MiaPaCa-2 cells, we identified among the top 10 enriched TFs not only NRF2 (NFE2L2) but also other TFs related to NRF2 activity including CEBPB, ATF3, and MAFF. Equally in BxPC3 cells, we identified NRF2 and some other TFs related to NRF2 activity including MAFG, ATF3, and BACH1. In line with these results, gene set enrichment analysis (GSEA) of the RNA-seq transcriptional profiling showed in both cell lines an upregulation of NRF2, UPR, and ferroptotic pathways in treated cells compared with control cells (**Figures 1C, D**, **Figures S1**, **S2**). To further detail the activation of NRF2 and ferroptosis-related genes, we searched in our RNA-seq data mRNAs (*p*-values smaller than 0.05) related to NRF2-dependent activation mechanism as well as genes related to ferroptosis. In particular, we identified six different cellular processes linked to NRF2 activity and/or ferroptosis, namely gluthatione metabolism, iron metabolism, ROS metabolism, lipid metabolism, metabolism, and autophagy as well as TFs associated with NRF2. As shown in the dendrograms presented in **Figure 2A**, several mRNAs linked to these selected processes were significantly regulated upon FR054 treatment, especially in MiaPaCa-2 cells compared to BxPC3 cells. Indeed, FR054 treatment transcriptionally upregulated most of the genes involved in GSH metabolism [*cystine/glutamate antiporters SLC7A11* and *SLC3A2* and *Glutathione-Disulfide Reductase (GSR)*]; glutamine, cysteine, and glycine metabolism (*GLS1*, *GCLM*, *GCLC*, *GOT1*, *PHGDH*, and *PSAT1*); NADPH production (*G6PD*, *PGD*, and *ME1*); and genes involved in anti-ferroptosis mechanisms such as iron transport and storage (*FTH1* and *FTL*), Fe2^+^ sequestration (*NUPR1* and *LCN2*), membrane repair mechanisms (*AIFM2*, *NQO1*, and *AKR1Cs*), and autophagic genes associated with both NRF2 activation and ferroptosis (*GABARAPL1*, *MAP1LC3B2*, and *SQSTM1*). Conversely, genes associated with lipid metabolism, generally repressed by NRF2, such as *ACACA*, *SCD*, *LPCAT3*, *FADS2*, and *ELOVL6* were downregulated, further confirming an FR054-dependent activation of the NRF2 axis. The latter effect was also confirmed by the concomitant upregulation of different TFs related to the NRF2 pathway including *MAFA*, *MAFG*, *MAFF*, *CEBPB*, *ATF4*, and *NRF2* as well. Of note, FR054 also induced a few important ferroptosis genes including *CHAC1*, a γ-glutamyl cyclotransferase toward glutathione, a well-known ferroptosis marker, and HMOX1, the major intracellular source of iron Fe^2+^ (**Figure 2B**). Several of these genes were equally regulated in BxPC3 but to a lesser extent, suggesting a lower antioxidant response as compared to MiaPaCa-2 cells upon FR054 treatment. Altogether, transcriptional data demonstrate that, in both cell lines, FR054 treatment is associated with the induction of genes involved in a large NRF2-dependent antioxidant response and a significant alteration of genes associated with either inhibition or induction of ferroptosis (**Figure 2B**), therefore suggesting that drugs interfering with these mechanisms could be used in the experiment of synthetic lethality in combination with FR054.

### Inhibition of glutamine/glutamate metabolism enhances the effect of FR054 by increasing PDAC cell death

NRF2 is a master regulator of antioxidative responses and plays critical roles in maintaining redox balancing; thus, NRF2 activators attenuate oxidative stress. Indeed, NRF2 is one of the key regulators of GSH metabolism. GSH synthesis enzyme glutamate-cysteine ligase, glutathione synthetase, and reductase are target genes of NRF2 (22). Glutaminase (GLS), which can catalyze glutamine into glutamate, is also promoted by NRF2 (23). Moreover, the expression of SLC7A11, the light chain subunit of the Xc- antiporter system (xCT), is activated by NRF2. xCT, a translocator of cystine and glutamate, imports and exports cystine and glutamate into and out of the cell, respectively. Since our transcriptional data clearly indicated an activation of the GSH metabolism upon FR054, we tested if inhibition of glutamine/glutamate utilization by PDAC cells could increase FR054 cytotoxicity, enlightening a cooperative lethal effect between HBP inhibition and GSH synthesis inhibition. To this end, MiaPaCa-2 cells were first treated for 24 h with FR054 (350 μM) and then treated for a further 24 h with two well-known inhibitors, ERA (10 μM), which blocks the activity of the antiporter SLC7A11, and Bis-2-(5-phenylacetamido-1,3,4-thiadiazol-2-yl)ethyl sulfide (BPTES) (10 μM), which blocks the activity of GLS1, either alone or in combination with FR054 (**Figure S3A** for the experimental scheme).

**Figure 3:**
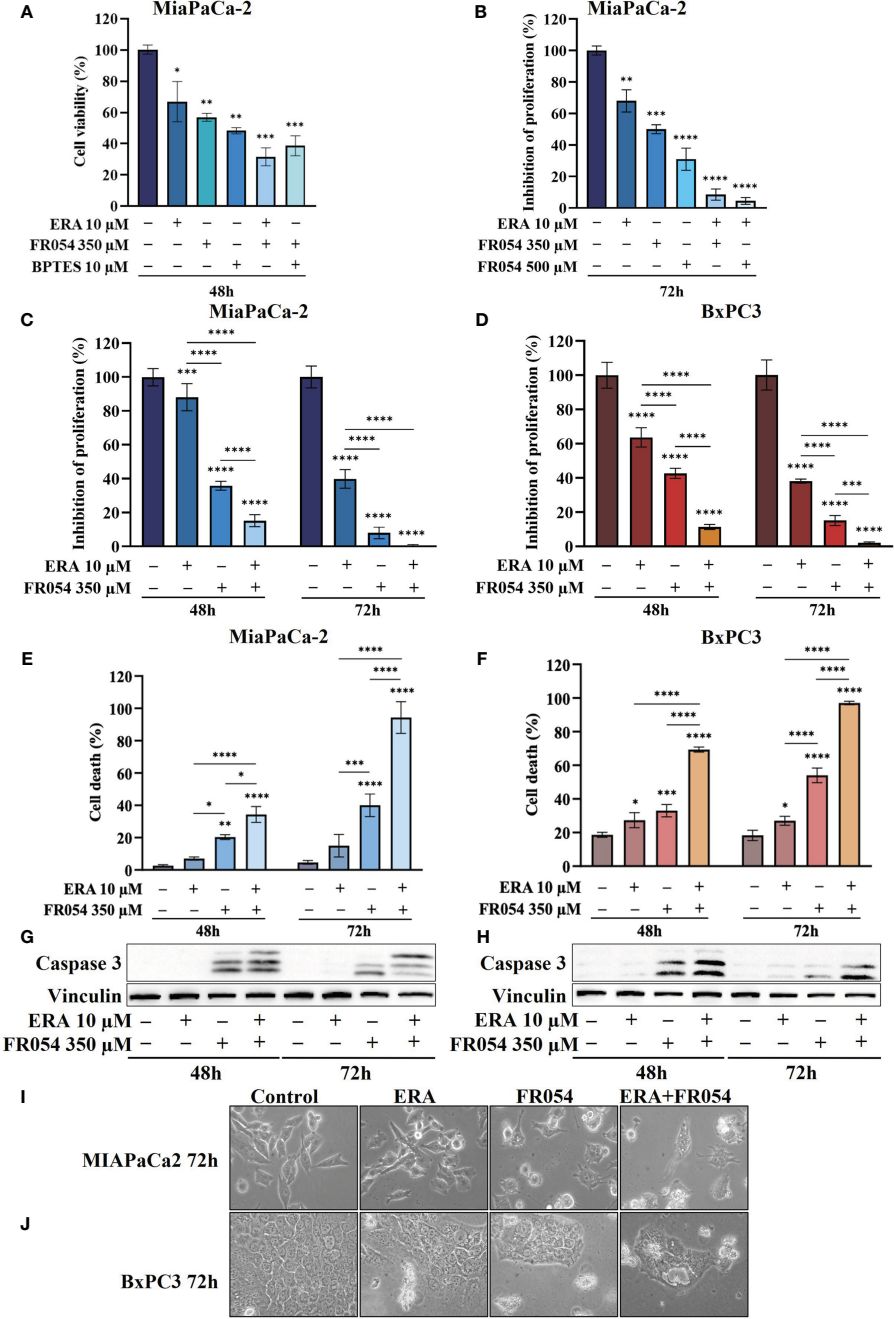
Combined treatment with FR054 and erastin enhances cell proliferation arrest and death of PDAC cell lines. **(A)** Trypan blue cell count after 48 h of treatment with FR054, erastin (ERA) 24 h, BPTES 24 h, or the combination of FR054 with ERA or BPTES added after 24 h of FR054 treatment. **(B)** Inhibition of proliferation expressed as percentage (trypan blue assay) after 72 h of treatment with two different doses of FR054 alone or in combination with ERA. **(C, D)** Inhibition of proliferation expressed as percentage (trypan blue assay) in MiaPaCa-2 and BxPC3 cells after 48 and 72 h of treatment with FR054 and ERA only or in combination. **(E, F)** Death cell percentage (trypan blue assay) in MiaPaCa-2 and BxPC3 cells after 48 and 72 h of treatment with FR054 and ERA only or in combination. **(G, H)** Western blot analysis of caspase 3 cleavage in cells treated as in **(C, D)**. Vinculine has been used as internal loading control. **(I, J)** Bright-field images of MiaPaCa-2 and BxPC3 cells treated as in **(C, D)** taken 48 h after the treatments. The data are presented as mean ± SD from three independent experiments. For **(A, B)**, statistical significance was determined using one-way ANOVA with Tukey’s multiple comparisons test, while for all the others, statistical significance was determined using two-way ANOVA with Tukey’s multiple comparisons test. *p < 0.05, **p < 0.01, ***p < 0.001 ****p < 0.0001.

In MiaPaCa-2 cells, as result of vital cell count, ERA treatment reduced cell proliferation by around 30%, BPTES by approximately 43%, and FR054 by approximately 52% compared to untreated cells (**Figure 3A**), suggesting an important proliferative role of glutamine metabolism in this cell line. Remarkably, the FR054 anti-proliferative effect was significantly enhanced when combined with ERA and BPTES since the proliferation reduction reached the values of 69% and 61% in combined treatments, respectively.

Taking into consideration the greater outcome of the combination of FR054 and ERA, we decided to further detail this effect by increasing the amount and the time of the treatment with FR054. In particular, the cells were treated as described in the scheme of **Figure S3B**. Briefly, cells were treated for 24 h with two different amounts of FR054 (350 and 500 μM) and then treated for a further 48 h with ERA (10 μM) alone or in combination with FR054. The cell survival was analyzed by counting the cells at 72 h. As shown in **Figure 3B**, 48 h of ERA treatment caused almost a 30% reduction in cell proliferation. As previously published, a dose-dependent cell proliferation arrest was observed in MiaPaCa-2 cells treated for 72 h with FR054 (12). Indeed, FR054 treatment at 350 and 500 μM caused a cell number reduction of approximately 36% and 70%, respectively, as compared to control cells (**Figure 3B**). Remarkably, such an anti-proliferative effect was significantly enhanced by ERA since the proliferation reduction reached 92% and 95% at 72 h in combined treatments, suggesting that SLC7A11 antiporter activity is necessary for cancer survival under HBP inhibition.

To further evaluate the cooperative effect of ERA when combined with FR054, we tested the combination in MiaPaCa-2 and BxPC3 cells along a time course of 72 h, by using the lowest dose of FR054 (350 μM) and analyzing both proliferation and cell death by vital trypan blue staining. As shown in **Figures 3C, D**, ERA alone at 48 h, as previously published (24), had a lower effect in MiaPaCa-2 cells as compared to BxPC3. Nevertheless, at 72 h, both cell lines induced almost 60% of cell proliferation arrest. FR054 had a stronger effect as compared to ERA, at both time points and in both cell lines. Strikingly, the combined treatment at 72 h induced approximately 100% of cell proliferation reduction, confirming the significant effect of this combination. The cytotoxic analysis additionally confirmed the above results (**Figures 3E, F**). Twenty-four hours and 48 h of ERA treatment, in both cell lines, induced a slight effect on cell death. Conversely, FR054 treatment (48 h and 72 h of treatment) was significantly more cytotoxic since cell death values were significantly higher at both time points. Strikingly, combined treatment, significantly enhanced cell death in both cell lines at both time points, causing almost 100% cell death at 72 h (**Figures 3E, F**), strongly confirming the effective cooperative outcome between FR054 and ERA in PDAC cells. As ERA is known for its pro-ferroptosis activity (25) and FR054 was shown to be able to induce apoptosis (11, 12), next we analyzed the activation/cleavage of caspase-3 as an exclusive apoptotic marker. At 48 and 72 h in FR054-treated cells as well as in combined treatment, caspase-3 signals increased as compared to control. However, despite the significant increase in cell death observed at 72 h (**Figures 3C, D**), caspase-3 cleavage was less pronounced as compared to 48 h, also suggesting a non-apoptotic mechanism of cell death (**Figures 3G, H**, **Figures S4**, **S5**). Conversely, in ERA alone at both time points, despite the increased cell death observed in particular in BxPC3 cells (**Figures 3C, D**), caspase-3 signal was not or barely detectable, confirming that the ERA was inducing a non-apoptotic cell death (**Figures 3G, H**, **Figures S4**, **S5**). Furthermore, cell morphological study further confirmed that combined treatment induced different morphological alterations as compared to single treatments (**Figures 3I, J**). Altogether, these findings support the notion that the combined treatment induces both an apoptotic and a non-apoptotic cell death.

### Cell death dependent on FR054 and ERA combination is associated with ER stress persistence and NRF2-dependent antioxidative response inactivation

Previously published data indicated that prolonged treatment with FR054 causes cell death by inducing ER stress and activation of UPR (11, 12). On the other hand, ERA treatment induces cysteine depletion and oxidative cell stress, causing both ER stress and then ferroptosis (26). Furthermore, transcriptional data shown in **Figure 2A** further suggest activation of UPR and of antioxidant response, especially in MiaPaCa-2 cells. Therefore, we decided to evaluate UPR activation and cellular oxidative stress upon single and combined treatments by immunoblot analysis.

**Figure 4:**
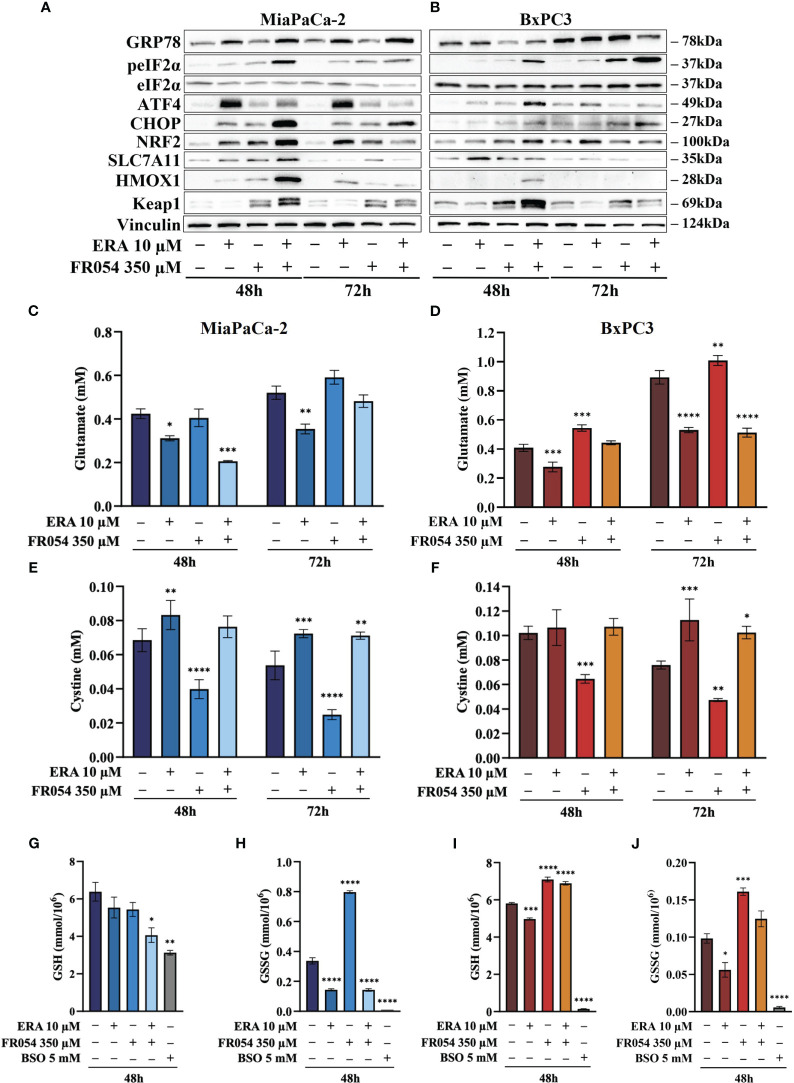
FR054 alone or in combination with erastin affects UPR and oxidative response protein expression, modifies SLC7A11 activity, and alters GSH and GSSG intracellular levels. **(A, B)** Immunoblot analysis showing the effects of the two compounds on different UPR and oxidative stress response proteins. **(C, D)** HPLC evaluation of the glutamate concentration in the growth medium of the MiaPaCa-2 and BxPC3 cells after the different treatments as indicated in the figure. **(E, F)** HPLC evaluation of the cystine concentration in the growth medium of the MiaPaCa-2 and BxPC3 cells after the different treatments as indicated in the figure. **(G, H)** Measurement of the intracellular concentration of GSH **(I)** and GSSG **(J)** in MiaPaCa-2 and BxPC3 cells after the different treatments as indicated in the figure. The data of GSH and GSSG concentration are presented as mean ± SEM from three independent experiments, while the other ones are presented as mean ± SD from three independent experiments. For **(C–E)**, and **(F)**, statistical significance was determined using two-way ANOVA with Tukey’s multiple comparisons test, while for **(G–J)**, statistical significance was determined using one-way ANOVA with Tukey’s multiple comparisons test. *p < 0.05, **p < 0.01 ***p < 0.001, ****p < 0.0001.

For UPR activation, we focused our analyses on eIF2α phosphorylation and protein expression of GRP78, linked to ER stress, ATF4, linked both to ER stress and cysteine depletion (27), and CHOP, a TF associated with cell cycle arrest (28) and induction of pro-apoptotic signals upon prolonged UPR activation (29). ERA treatment of MiaPaCa-2 cells, at both 48 and 72 h, resulted in an increased expression of GRP78, ATF4, and CHOP as compared to control cells (**Figure 4A**, **Figure S4**). No significant change in eIF2α phosphorylation was observed. FR054 treatment resulted in an increase of eIF2α phosphorylation, a slight increase of ATF4 at both 48 h and 72 h, and a more significant increase of CHOP as compared to control cells. No significant change in GRP78 expression was observed (**Figure 4A**, **Figure S4**). Of note in FR054-treated cells, ATF4 was induced at significantly lesser extent as compared to ERA treatment. Combined treatment resulted in higher levels of eIF2α phosphorylation, GRP78, and CHOP as compared to control and single drug-treated cells. ATF4 was induced at 48 h to significantly decrease at 72 h. Nevertheless, ATF4 level was significantly lower as compared to ERA-treated cells (**Figure 4A**, **Figure S4**). Next, we analyzed the behavior of the same proteins in BxPC3 cells, characterized by the expression of a wild-type K-Ras and known to be more sensitive to ferroptosis inducers. As shown in **Figure 4B**, **Figure S5**, in BxPC3 cells treated with ERA, the different UPR markers were generally upregulated but in no significant manner (eIF2α phosphorylation, ATF4, and CHOP) or downregulated in a significant manner (GRP78). FR054 treatment resulted in a time-dependent increase of eIF2α phosphorylation and CHOP, in a nonsignificant increase of ATF4 and a significant downregulation of GRP78 at 48 h (**Figure 4A**, **Figure S4**). Conversely, in cells treated with the combination, the three UPR markers, eIF2α phosphorylation, GRP78, and CHOP, increased significantly in a time-dependent manner, especially at 72 h, as compared to control and single-treated cells (**Figure 4B**, **Figure S5**). Of note in combined treatment, ATF4 expression, conversely to what was observed in MiaPaCa-2 cells, increased significantly only at 48 h to decrease at 72 h. Taken together, our findings, in line with previous reports showing the protective role of ATF4 (30, 31), could suggest that the ERA resistance of MiaPaCa-2 cells as compared to BxPC3 cells may be ascribed to a higher ATF4 level of expression as compared to BxPC3 cells and that such resistance is hindered by the ability of combined treatment to induce UPR, as highlighted by increased eIF2α phosphorylation as well as GRP78 and CHOP higher expression, in tight association with ATF4 decrease. Indeed, in the combination-treated cells, the sample in which we observed the highest level of cell death (**Figure 3C**), CHOP remained always higher as compared to ATF4. Such a protective role of ATF4 is further suggested by BxPC3 results, in which the highest values of cell death observed in combination-treated cells (**Figure 3D**, 72 h) were associated with low and high levels of ATF4 and CHOP, respectively. Previous transcriptional data indicated that FR054 treatment can transcriptionally induce an NFR2-dependent antioxidant response, proposing that FR054, as hitherto shown in breast cancer cells (11), induces cellular oxidative stress in PDAC cells. Since several data indicated that NRF2 and ATF4 interact and cooperatively upregulate a number of antioxidant and antiapoptotic genes involved in a protective response engaged during ER stress (32, 33), we decided to determine NRF2 expression under our experimental conditions. As expected, after 48 h, the protein level of NRF2 was upregulated in response to ERA and FR054 and such upregulation was stronger in combined treatment in both cell lines (**Figures 4A, B**, **Figures S4**, **S5**), indicating increased oxidative stress. Surprisingly, at 72 h, while NRF2 expression remained high (MiaPaCa-2 cells) or was further induced (BxPC3 cells) in ERA-treated cells, conversely in FR054-treated samples, especially upon combined treatment, it was significantly reduced. This behavior was observed especially in ERA-resistant MiaPaCa-2 cells, further suggesting that ATF4 and NRF2 levels may dictate ERA resistance (**Figures 4A, B**, **Figures S4**, **S5**). Oxidative stress as well as UPR, stabilizing and activating NRF2, regulates downstream gene transcription, including SLC7A11 and HMOX1 (34–36). The former amplifies glutamate secretion and cystine uptake, and facilitates GSH synthesis for reactive oxygen species (ROS) detoxification (37), while the latter helps keep the cellular redox balance by catalyzing the degradation of heme to carbon monoxide, iron, and biliverdin (38). Thus, to demonstrate NRF2 activation, we analyzed SLC7A11 and HMOX1 protein levels. As shown in **Figure 4A**, **Figure S4**, in MiaPaCa-2 cells, both SLC7A11 and HMOX-1 almost mirrored NRF2 expression at both time points. Conversely, in BxPC3 cells, both proteins were less modulated and only partially mirrored NRF2 expression, since both were only upregulated at 48 h and in particular SLC7A11 slightly increased only in ERA-treated cells and HMOX1 only in combined-treated cells. Nevertheless, both proteins significantly decreased at 72 h (**Figure 4B**, **Figures S4**, **S5**), further suggesting that the major sensitivity of BxPC3 cells to ERA and combined treatments may be ascribed to low ATF4 expression and NRF2 activation. To explore the oscillatory expression of NRF2 protein upon single and combined treatment, we then determined the expression level of *Kelch-like ECH-associated protein 1* (KEAP1), a well-known negative regulator of NRF2 protein stability (39). The expression of KEAP1 significantly increased only in FR054-treated cells and at 48 h since at a later timepoint the level decreased, especially in combined treatment (**Figure 4A** and **Figures S4**, **S5**), therefore justifying the reduced NRF2 expression in these cell samples. Altogether, these data suggest that increased cell death in combined treatment (**Figures 3E, F**) is tightly linked to a significantly higher expression of the pro-apoptotic protein CHOP and a greater antioxidative response at an early time point, as confirmed by the higher NRF2, SLC7A11, and HMOX1 levels, which, at a later time point, together with ATF4, abruptly decrease.

### FR054 modulates SLC7A11 function without restraining the inhibitory effect of erastin

To better understand the relation between the observed level of SLC7A11 protein and its function as an exchanger of the anionic form of cystine and glutamate, we measured the amount of these two amino acids into cultured media in both cell lines. While ERA, as expected, inhibited glutamate release in both cell lines and at both time points analyzed, FR054 alone, as predicted by the transcriptional data, albeit not statistically significant, caused a time-dependent increase in the amount of released glutamate (**Figures 4C, D**). Of note, despite the different temporal dynamics, ERA combined with FR054 still inhibited glutamate release in both cell lines counteracting the positive effect of FR054 treatment (**Figures 4C, D**). Since glutamate is secreted in exchange with cystine uptake, necessary for GSH synthesis, we analyzed extracellular levels of cystine. Both lines treated with ERA alone or in combination with FR054 showed a cystine uptake reduction as compared to control cells, mirroring the effect on glutamate efflux (**Figures 4E, F**). Conversely, in FR054-treated cells, a significant reduction of extracellular cystine was observed, confirming the ability of FR054 to increase the exchange between glutamate and cystine (**Figures 4E, F**). The latter result was also confirmed in BxPC3 cells treated with FR054 alone given that in this cell line, there was also a significant decrease of extracellular cystine (**Figure 4F**). Given that one molecule of cystine can then be converted into two molecules of cysteine, which is a committed step for GSH biosynthesis next, we evaluated at 48 h the intracellular level of GSH and GSSG. As technical control, we treated the cells for 24 h with 5 mM buthionine sulfoximine (BSO) alone, an inhibitor of GCLC activity. GSH in MiaPaCa-2 cells was statistically significantly reduced only in cells treated with the combination of the two drugs as compared to control cells. Indeed, in ERA- and FR054-treated cells, GSH still decreased but not in a statistically significant value (**Figure 4G**). Conversely, a significant variation of GSSG levels was observed in all treated cells as compared to the control (**Figure 4H**). In fact, in cells treated with ERA alone or in combination with FR054, in accordance with the significant reduction of cystine uptake, the GSSG levels were also drastically reduced (**Figure 4H**). A similar behavior was observed in BSO-treated cells, in which the prolonged inhibition of GCLC caused a significant decrease of both GSH and GSSG, as previously observed (40). Surprisingly, in FR054-treated cells, consequently to the significant cystine uptake, there was a significant accumulation of GSSG (**Figure 4H**). Analysis of GSH and GSSG levels in BxPC3 as compared with the control group showed that ERA induced a slight but significant reduction in both GSH and GSSG levels (**Figures 4I, J**). Conversely, in FR054-treated cells, either alone or in combination with ERA, both GSH and GSSG levels remained higher as compared to control cells (**Figures 4I, J**). Of note, BxPC3 cells were more sensitive to BSO treatment since 24 h treatment completely depleted both GSH and GSSG. Such a result in combined treatment was in part unexpected because following this treatment a significant reduction in extracellular glutamate as well as in cystine uptake was observed. However, based on our transcriptional data (**Figure 2A**), we cannot exclude the role of the trans-sulfuration pathway in intracellular synthesis of cysteine given that the *Cystathionine Beta-Synthase* (*CBS*) mRNA was significantly upregulated only in this cell line upon FR054 treatment. Altogether, these findings suggest that FR054 alone increases in both cell lines SLC7A11 glutamate/cystine exchange and intracellular GSSG level. Conversely, when combined with ERA, MiaPaCa-2 cells might promote cell death by reducing glutamate/cystine exchange and GSH synthesis, while in BxPC3, its proapoptotic effect, despite being linked to a significant reduction of glutamate/cystine exchange, does not appear to be related to GSH level reduction.

### The combined effect between FR054 and erastin on cell survival is associated with oxidative imbalance, causing lipid peroxidation

**Figure 5:**
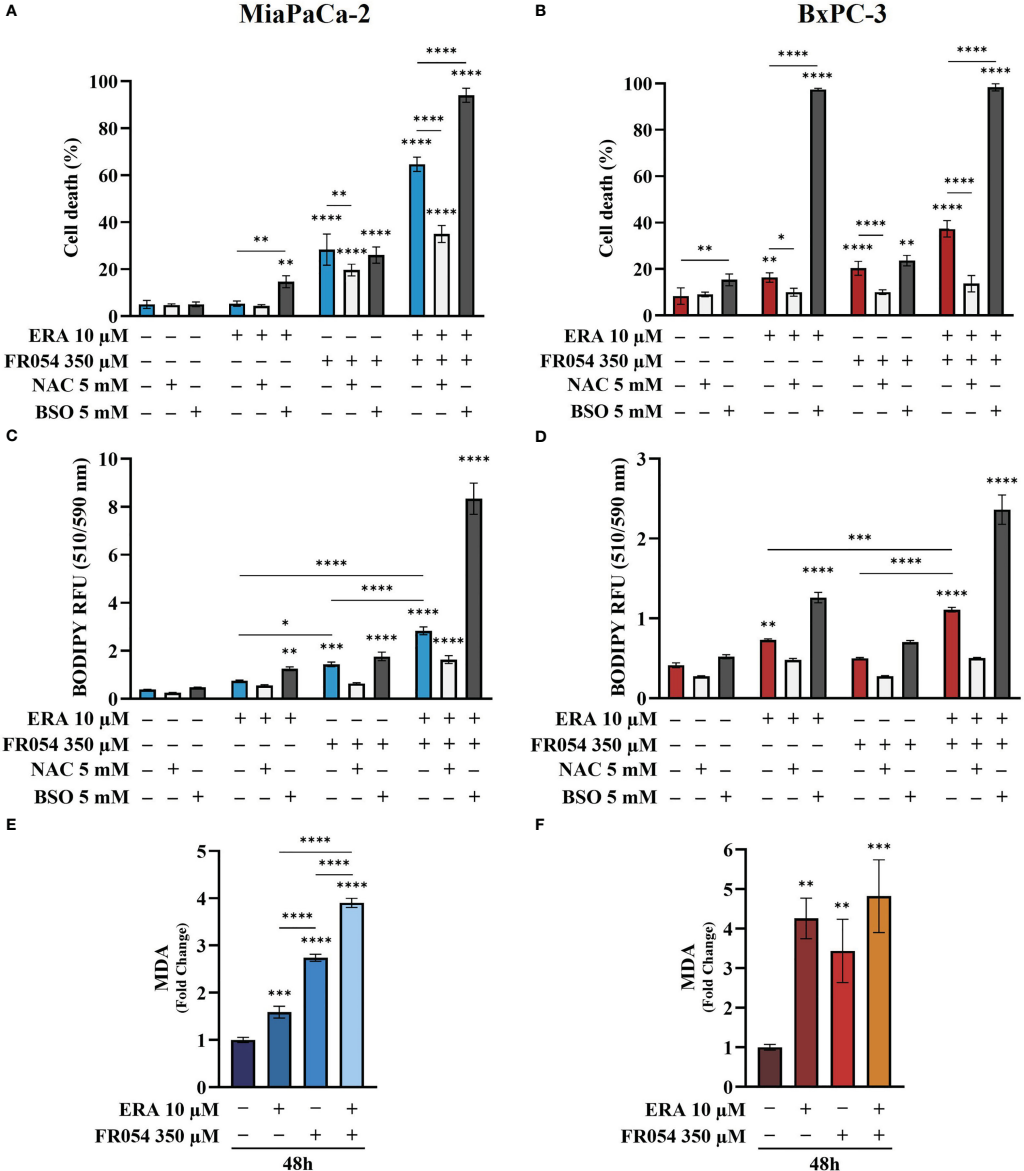
Variation of intracellular level of GSH by using NAC or BSO preserves or impairs, respectively, PDAC cell vitality by decreasing or increasing lipid peroxidation in PDAC cells. **(A, B)** Death cell percentage data (trypan blue assay) of MiaPaCa-2 and BxPC3 cells after the indicated treatments. **(C, D)** Intracellular lipid-ROS levels in MiaPaCa-2 and BxPC3 cells after the indicated treatments. **(E, F)** Malondialdehyde (MDA) assay to evaluate the lipid peroxidation in MiaPaCa-2 and BxPC3 cells after the indicated treatments. The death cell percentage data and MDA assay data are presented as mean ± SD from three independent experiments, while intracellular lipid-ROS data are presented as mean ± SEM from three independent experiments. For **(A–D)**, statistical significance was determined using two-way ANOVA with Tukey’s multiple comparisons test, while for **(E, F)**, statistical significance was determined using one-way ANOVA with Tukey’s multiple comparisons test. *p < 0.05, **p < 0.01 ***p < 0.001, ****p < 0.0001.

To evaluate the effect of intracellular GSH modulation and oxidative stress on PDAC cell survival under single and combined treatments with ERA and/or FR054, we used, as schematized in **Figure S3C**, two different approaches: 24 h of cotreatment with *N*-acetyl-L-cysteine (NAC, 5 mM), a cysteine prodrug, able to replenish intracellular GSH levels, or 24 h of cotreatment with BSO (5 mM) to block *de novo* synthesis of GSH. NAC cotreatment, as measured by trypan blue vital assay, led to decreased cell death in both cell lines and in all the conditions analyzed as compared to control cells (**Figures 5A, B**). Conversely, cotreatment with BSO led to increased cell death in cells treated with ERA alone or in combination with FR054 (**Figures 5A, B**). Of note, in FR054-treated samples, in which previous data indicated a significant enhancement of SLC7A11 activity tightly associated with a great accumulation of GSSG, suggesting a more functionally GSH biosynthetic pathway (**Figures 2** and **4**), the effect of BSO was negligible.

Based on our transcriptional data, revealing a role of ferroptosis in PDAC cells after FR054 treatment, given the significant effect of FR054 on NRF2 pathway activation as well as the noteworthy impact of BSO on PDAC cell survival when combined with ERA alone or in combination with FR054, we next sought to measure lipid peroxidation (lipid ROS) at 48 h as a hallmark of cystine depletion as well as ferroptosis by using the fluorescent probe C11-BODIPY. As expected from previous results, treatment with ERA or FR054 alone increased fluorescence, especially in ERA-sensitive BxPC3 and slightly in FR054-treated MiaPaCa-2 cells. Conversely, combined treatment induced a significant increase in fluorescence in both cell lines (**Figures 5C, D**). As expected by cell survival data, NAC and BSO treatments reduced and increased lipid ROS, respectively, especially in cells treated with the drug combination, suggesting that these cells are more sensitive to intracellular redox state. To further demonstrate lipid peroxidation, we measured the MDA, an end product of lipid peroxides, generated during ferroptosis and other oxidative stress context. As shown in **Figures 5E, F**, in both cell lines, MDA levels almost resembled C11-BODIPY staining, given the increase upon all treatments, especially in combined treated samples. Thus, these findings indicate that FR054, increasing cell dependence for the SLC7A11–GSH axis, causes a significant increase in Lipid-ROS when combined with ERA, and this effect occurs in both cell lines.

### *Stable-isotope-assisted metabolomics analysis of central carbon metabolism indicates that the* FR054 enhancing effect on ferroptosis is associated with a reduction of glucose entry into the TCA cycle and an increased glutaminolysis

**Figure 6:**
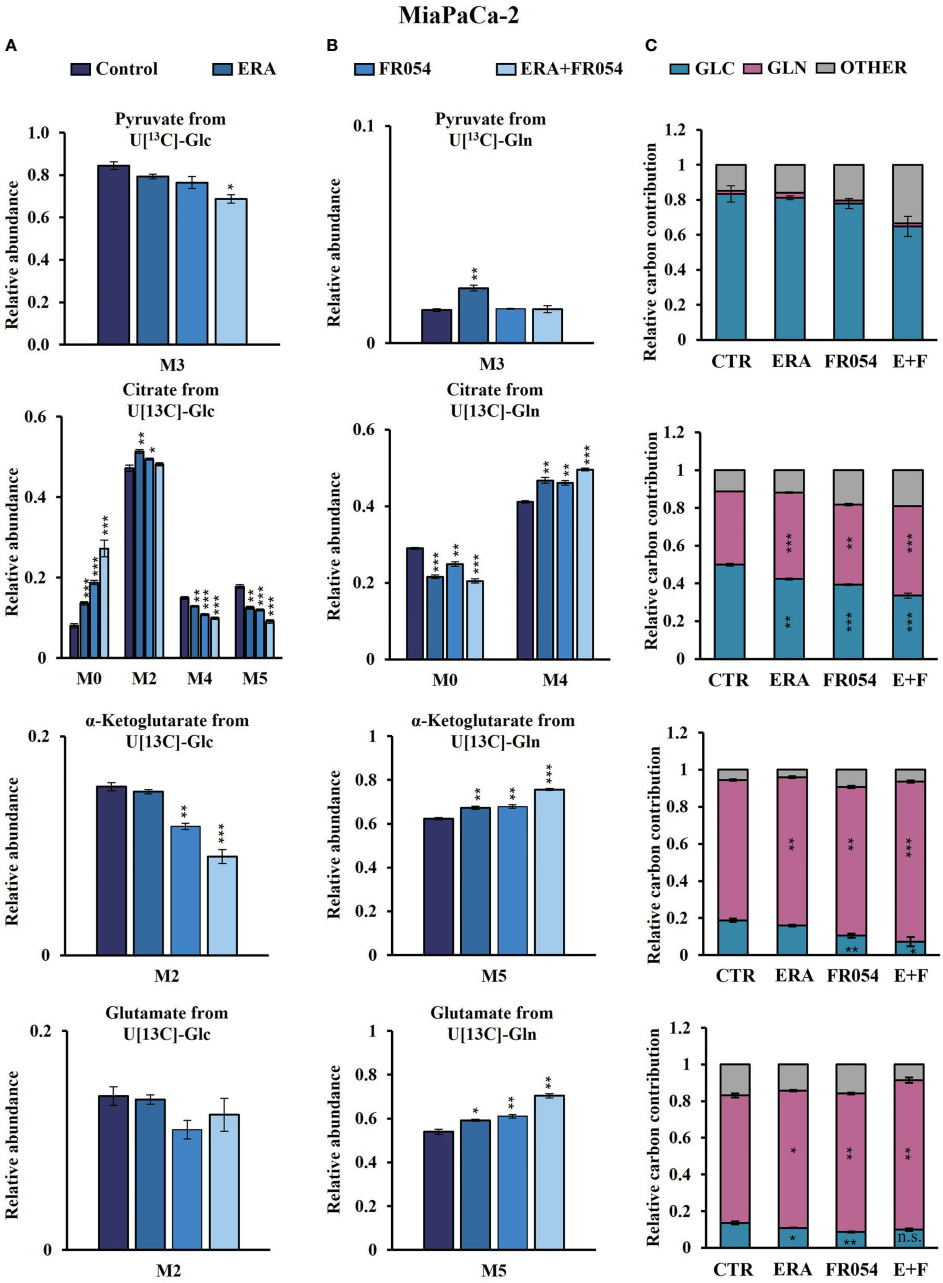
Combined treatment in MiaPaCa-2 cells favors glutaminolysis over glycolysis. **(A, B)** 13C labeling of pyruvate, citrate, a-ketoglutarate, and glutamate from MiaPaCa-2 cells incubated in [U-^13^C_6_]-glucose or [U-^13^C_5_]-glutamine medium for 48 h. **(C)** Relative carbon fractional contribution of glucose and glutamine to pyruvate, citrate, a-ketoglutarate, and glutamate formation. The data are presented as mean ± SEM from three independent experiments. The statistical significance was performed with Student’s *t*-test. *p < 0.05, **p < 0.01, ***p < 0.001.

**Figure 7:**
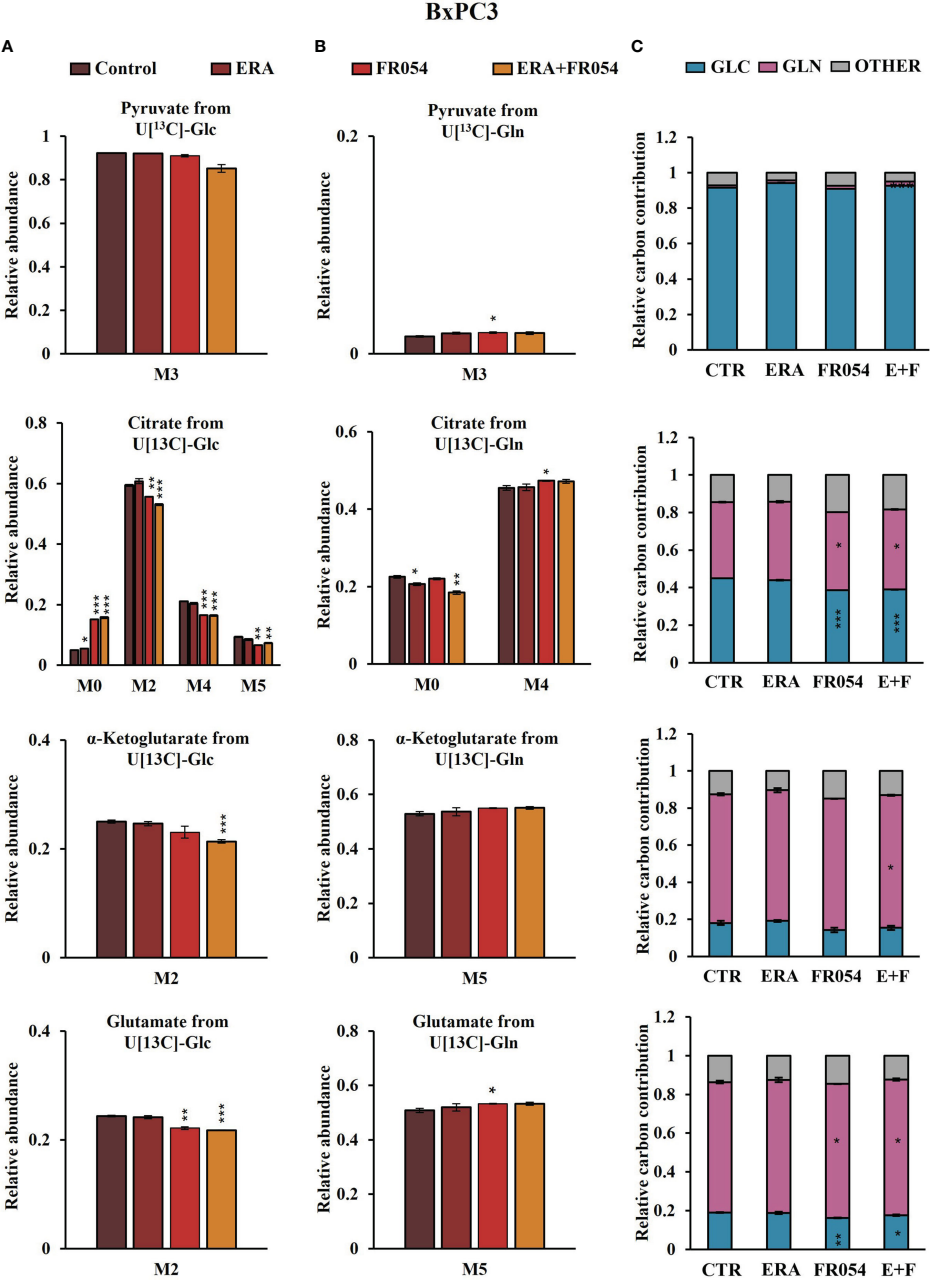
Combined treatment in BxPC3 cells reduces glucose utilization through the TCA cycle and slightly increases glutaminolysis. **(A, B)** ^13^C labeling of pyruvate, citrate, a-ketoglutarate, and glutamate from BxPC3 cells incubated in [U-^13^C_6_]-glucose or [U-^13^C_5_]-glutamine medium for 48 h. **(C)** Relative carbon fractional contribution of glucose and glutamine to pyruvate, citrate, a-ketoglutarate, and glutamate formation. The data are presented as mean ± SEM from three independent experiments. The statistical significance was performed with Student’s *t*-test. *p < 0.05, **p < 0.01, ***p < 0.001.

To further investigate the underlying basis for the role of FR054 in increasing ERA sensitivity, we conducted a stable-isotope-assisted metabolomics analysis comparing all cell lines after the different treatments as described in **Figure S3D**. Metabolic analysis was performed by labeling the different cell lines with either [U-^13^C_6_]-glucose or [U-^13^C_5_]-glutamine and quantified isotopic enrichment in the form of MIDs. To analyze glycolysis, TCA cycle, and glutaminolysis, we analyzed the glucose or glutamine carbon contribution to the generation of pyruvate, citrate, α-ketoglutarate, and glutamate. In both cell lines, we observed a significant reduction of M4 and M5 citrate when cells were treated with FR054 alone (MiaPaCa-2, M4: −28%, M5: −33%; BxPC3, M4: −22%, M5: −29%) or in combination with ERA (MiaPaCa-2, M4: −34%, M5: −49%; BxPC3, M4: −22%, M5: −22%), suggesting a reduced activity of both pyruvate dehydrogenase (PDH) and pyruvate carboxylase (PC) (**Figures 6A**, **7A**, **Figure S6A**, **Supplementary Table 4**). We also observed this reduction in the M2 of α-ketoglutarate when cells were treated with FR054 alone (MiaPaCa-2, M2: −24%; BxPC3, M2: −8%) or in combination with ERA (MiaPaCa-2, M2: −41%; BxPC3, M2: −15%) (**Figures 6A**, **7A**, **Supplementary Table 4**), a key TCA cycle intermediate, pointing towards an overall reduction of glucose entry into the TCA cycle. Indeed, in both cell lines, we observed a significant decrease in the glucose carbon contribution for their synthesis (**Figures 6C**, **7C**). The effect of FR054, however, was much stronger in MiaPaCa-2 cells compared to BxPC3. Decreased glucose contribution suggests that cells were more dependent on glutamine for TCA cycle anaplerosis. In fact, analysis of the same metabolites in cells labeled with [U-^13^C_5_]-glutamine indicated significantly higher enrichment of M5 glutamate (MiaPaCa-2, ERA: 10%, FR054: 13%; ERA+FR054: 30%; BxPC3, ERA: 2%, FR054: 5%; ERA+FR054: 5%), M5 α-ketoglutarate (MiaPaCa-2, ERA: 8%, FR054: 9%; ERA+FR054: 21%; BxPC3, ERA: 1%, FR054: 4%; ERA+FR054: 4%), and M4 citrate (MiaPaCa-2, ERA: 14%, FR054: 12%; ERA+FR054: 20%; BxPC3, ERA: 0%, FR054: 4%; ERA+FR054: 4%) upon all treatments and especially in the combined treatment (**Figures 6B**, **7B**, **Figure S6B**, **Supplementary Table 4**), suggesting that FR054 enhances glutaminolysis, as confirmed by the significant increase of relative glutamine carbon contributions for their synthesis (**Figures 6C**, **7C**). Similar to the [U-^13^C_6_]-glucose results, the effect was much stronger in MiaPaCa-2 cells compared to BxPC3. An increase in labeled M3 pyruvate was observed only in ERA-treated samples in MiaPaCa-2 cells, suggesting only a slight alteration of malic enzyme activity in ERA-treated cells (**Figure 6B**). Nonetheless, analysis of the glutamine carbon contribution confirmed that in FR054-treated BxPC3 cells, glutamine becomes more relevant but to a lesser extent as compared to K-Ras mutated MiaPaCa-2 cells (**Figure 7C**). Altogether, metabolomics analysis suggests that in both cell lines, treatment with FR054 alone or in combination, with a different magnitude, reduces glucose utilization through the TCA cycle. Here, we report that especially in MiaPaCa-2 cells, FR054 treatment significantly enhances cell dependence on glutamine and glutaminolysis, in line with previous findings describing glutaminolysis as a mechanism to increase ferroptosis cell death (41).

### Combined treatment between ERA and FR054 induces enhancement of ferroptosis in PDAC cell lines independent on K-Ras mutation

**Figure 8:**
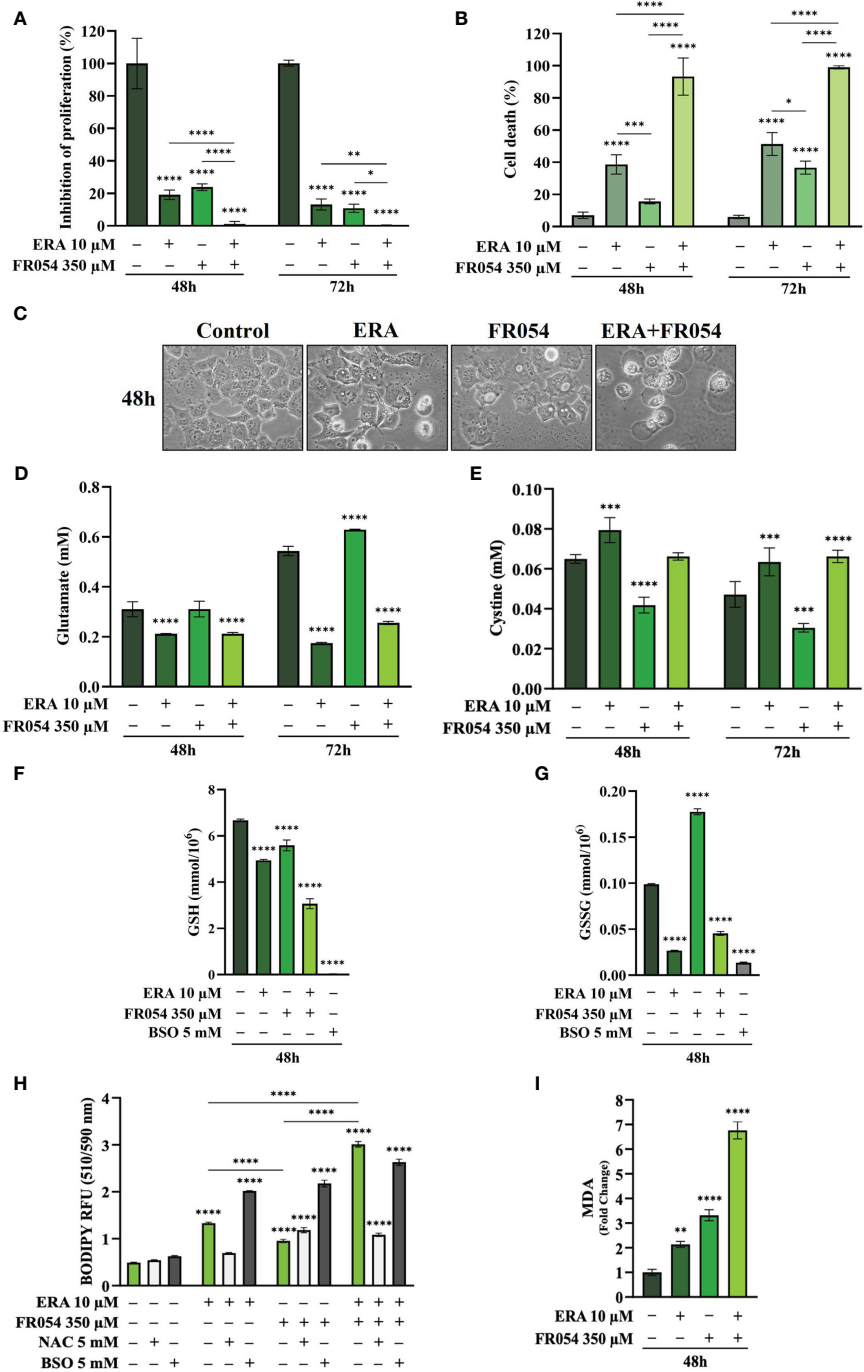
Combined treatment with FR054 and erastin enhances cell proliferation arrest and death of PANC1 cells by interfering with SLC7A11 activity and GSH metabolism and causing lipid peroxidation. **(A)** Trypan blue cell count in percentage after 48 h and 72 h of treatment with FR054, erastin (ERA) 24 h or 48 h, or their combination. **(B)** Cell death expressed as percentage (trypan blue assay) after 48 and 72 h of treatment with FR054 alone or in combination with ERA. **(C)** Bright-field images of PANC1 cells treated as **(A)** taken 48 h after the treatment. **(D, E)** HPLC evaluation of glutamate and cystine growth medium concentration, respectively, after the different treatments as indicated in the figure. **(F)** GSH and **(G)** GSSG intracellular analysis upon the different treatments as indicated in the figure. **(H)** Intracellular lipid-ROS levels in PANC1 cells after the indicated treatments. **(I)** Malondialdehyde (MDA) assay to evaluate the lipid peroxidation in PANC1 cells after the indicated treatments. The data are presented as mean ± SD from three independent experiments. Only the data of GSH and GSSG concentration, and the data of lipid-ROS levels are presented as mean ± SEM from three independent experiments. For **(A–E)**, and **(H)**, statistical significance was determined using two-way ANOVA with Tukey’s multiple comparisons test, while for **(F)**, **(G)**, and **(I)**, statistical significance was determined using one-way ANOVA with Tukey’s multiple comparisons test. *p < 0.05, **p < 0.01, ***p < 0.001 ****p < 0.0001.

To further reinforce the notion that ERA and FR054 treatment could cooperate in inducing ferroptosis in PDAC cell models, we treated human pancreatic cancer PANC1 cells, characterized by K-Ras mutation and a great sensitivity to cysteine depletion, with ERA and FR054 alone or in combination along a time course of 72 h. We observed a significant effect of all treatments on proliferation (**Figure 8A**) and a time-dependent effect on cell death (**Figure 8B**). In both types of analysis, the combinatorial treatment induced the stronger outcome as confirmed also by morphological cell analysis, in which cell swelling before cell disruption was observed (**Figure 8C**). Next, we analyzed glutamate and cystine levels, confirming that ERA alone or in combination significantly inhibited SLC7A11 function (**Figures 8D, E**) and FR054 alone induced a significant increase in SLC7A11 function. To evaluate the downstream effects of SLC7A11 modulation by the different treatments, we measured GSH and GSSG levels. The effect of all treatments significantly reduced GSH levels, and such reduction was much stronger compared to MiaPaCa-2 and BxPC3 cells (**Figure 8F**). Of note, GSSG levels were significantly modulated by all treatments and to the same extent as in MiaPaCa-2 cells, further confirming the ability of ERA and FR054 to decrease or increase GSSG level, respectively (**Figure 8G**). Then, we examined whether, upon all treatments and adding NAC or BSO, PANC1 cells promoted lipid ROS and MDA generation. Single and combined treatments significantly increased C11-BODIPY fluorescence and intracellular MDA (**Figures 8H, I**). NAC and BSO treatment, as previously observed for the other two PDAC cell lines, significantly reduced or increased C11-BODIPY fluorescence, suggesting that, also in PANC1 cells, the combined treatment enhanced ferroptosis. Remarkably, our findings confirmed the great sensitivity of PANC1 cells to ERA treatment, as previously described, a sensitivity that was further enhanced by FR054. Thus, the enhancer role of FR054 in ferroptosis could be attributed to the boosting of lipid peroxidation.

To further dissect the mechanism by which FR054 increased ERA sensitivity in PANC1 cells as compared to two other PDAC cell lines, we also conducted in PANC1 a stable-isotope-assisted metabolomics analysis as previously described. In PANC1 cells, we observed a significant reduction of M4 (ERA: −11%, FR054: −8%; ERA+FR054: −23%) and M5 citrate (ERA: −26%, FR054: −17%; ERA+FR054: −54%) when cells were treated with ERA and FR054 alone or in combination, suggesting a reduced activity of both PDH and PC (**Figure S7A**, **Supplementary Table 4**). We also observed this reduction in the M2 of α-ketoglutarate (ERA: −7%, FR054: −3%; ERA+FR054: −23%) and glutamate (ERA: −7%, FR054: −4%; ERA+FR054: −25%) (**Figure S7A**), further suggesting an overall reduction of glucose entry into the TCA cycle as demonstrated also by the significant decrease in the glucose carbon contribution for their synthesis (**Figure S7C**). Next, we analysed the same metabolites in PANC1 cells labeled with [U-^13^C_5_]-glutamine. A significant higher enrichment of M5 glutamate (ERA: 39%, FR054: 13%; ERA+FR054: 68%), M5 α-ketoglutarate (ERA: 36%, FR054: 12%; ERA+FR054: 62%), and M4 citrate (ERA: 35%, FR054: 10%; ERA+FR054: 54%) upon all treatments and especially in combined treatment was observed (**Figure S7B**, **Supplementary Table 4**), suggesting that the combined treatment enhances glutaminolysis, as confirmed by the significant increase of relative glutamine carbon contributions for their synthesis (**Figure S7C**). Altogether, metabolomics analysis indicated also that in PANC1 cells, as observed in MiaPaCa-2 cells, the combined treatment reduces glucose utilization through the TCA cycle and significantly enhances cell dependence on glutamine and glutaminolysis, suggesting a role of glutaminolysis in ferroptosis induced by the combined treatment.

## Discussion

Previous studies suggested that PDAC exhibits extensive metabolic reprogramming necessary to support survival and growth and that such metabolic changes could represent new possible therapeutic targets (42). On the other hand, it has been recently shown that cysteine depletion induces PDAC cell death through a process named ferroptosis, a novel mechanism of non-apoptotic cell death, recently identified as another promising tumor therapeutic strategy (24). In this study, we investigated the anticancer effect of FR054, an inhibitor of the metabolic route named HBP, found altered in PDAC (6, 12, 13, 43), in combination with ERA, a specific inhibitor of the antiporter SLC7A11, able to reduce cysteine uptake, and hence to stimulate ferroptosis. We show that such a combination, tested for the first time to the best of our knowledge, induces a significant enhancement of the ERA effect causing massive cell death, the latter characterized by both apoptosis and ferroptosis. Of note, we show that the combination stimulates, as compared to single treatments, an ER stress-dependent mechanism of cell protection, namely, the ATF4–NRF2–SLC7A11–GSH axis, which causes an increased dependence on SLC7A11 activity, whose inhibition with ERA leads to massive cell death. Of note, although recent reports indicate both positive and negative association between ER stress and ferroptosis (44, 45), the mechanism supporting a contribution of ER stress in ferroptosis remains to be further clarified.

Here, by using RNA-seq data and GSEA, we identified that UPR activation, due to HBP inhibition, stimulates a wide transcriptional response involving several key regulators of cellular antioxidative response including the TFs NRF2, which lies at the center of a complex regulatory network governing cellular oxidative stress. Indeed, we identified several mRNAs involved in GSH synthesis, ROS detoxification, iron metabolism, and metabolic enzymes all upregulated especially in oncogenic K-Ras expressing MiaPaCa-2 cells as compared to wild-type expressing K-Ras BxPC3 cells. Such antioxidant response activated by UPR has been already described; indeed, accumulation of unfolded proteins causes an internal ER oxidative stress at which ER reacts by the activation of a complex response among which is the induction of NRF2 (46). Importantly, cellular oxidative stress is able to activate ER stress as well (47). In addition, UPR modulates autophagy and ROS metabolism. More recent findings indicate that it may have an important impact also on cellular iron homeostasis, i.e., by modulating the gene expression of ferroportin and ferritin (48). Therefore, in line with our previous findings (11, 12), we can assume that FR054, interfering with protein folding, is a potent UPR inducer, as confirmed by our transcriptional data in which we observed upregulation of some UPR targets in both cell lines such as DDIT3 (CHOP), ATF4, ATF3, and CHAC1 (**Figure 2**). Intriguingly our transcriptional analysis showed that FR054 treatment caused a rather contrasting effect since we observed a significant upregulation of several mRNAs encoding for proteins involved in GSH metabolism such as SLC7A11 and HMOX1, the latter being an ER-resident protein, whose expression has been related to the induction of ferroptosis (49, 50).

Based on these findings and considering that ferroptosis can be exploited and then applied in clinical treatment strategies, we reasoned that a ferroptosis inducer, ERA, could significantly enhance the effect of FR054 in stimulating cell death. Indeed, in all the PDAC cell lines described in the manuscript, independently of their mutation status in K-Ras protein, the combined treatment as compared to single treatment caused an earlier cell proliferation arrest and then almost 100% cell death. Of interest, analysis of the pro-apoptotic protein CHOP confirmed its expression in both ERA- and FR054-treated cell samples, therefore substantiating a possible apoptotic mechanism activated by the single and combined treatments. On the other hand, another key factor involved in UPR response is ATF4. Previous studies have demonstrated that ATF4 has a protective role against ferroptosis or amino acid deprivation in cancer cells (26, 30, 51). However, other studies also indicated that ATF4 may contribute to cell death under the same types of stress (51–53). Therefore, ATF4 has a dual role in cell death, and consequently, its final effect as a regulator of ferroptosis appears to be cell context-dependent.

Here, we demonstrated that in ERA resistant-MiaPaCa-2 cells, ERA treatment was able to induce the UPR, as confirmed by increased GRP78 and CHOP expression, a pro-survival anti-oxidative response, as confirmed by NRF2 enhanced expression and above all a high level of ATF4, imputable to cystine deprivation. Conversely, FR054 in the same cells also induced UPR and the anti-oxidative response but not a comparable level of ATF4, since no cystine depletion was observed upon this treatment. Strikingly, MiaPaCa-2 cells treated with the combination showed at an early time point (48 h) a significant stronger activation of UPR and of the anti-oxidative response but a lower level of ATF4 as compared to ERA. However, at a later time point (72 h), only the UPR remained significantly active as the anti-oxidative response as well as ATF4 significantly declined. On the other hand, the regulation of the same proteins in BxPC3 cells indicated their different behavior following the different treatments. Indeed, in ERA- and FR054-treated cells, the UPR, ATF4, and anti-oxidative responses were slowly activated and in a minor measure as compared to MiaPaCa-2 cells. Nonetheless, also in this cell line, the combined treatment induced a persistent UPR and a significant decrease of the anti-oxidative response as well as of ATF4, suggesting a minor cysteine depletion, as we observed, and slower intracellular oxidative stress. From these data, we can argue that the lower sensibility to ERA of MiaPaCa-2 cells as compared to BxPC3 cells, as previously described (24), must be probably ascribed to their ability to induce an ATF4-dependent response that together with NRF2 activation is able to avoid the cytotoxic effect of ERA. Instead, in BxPC3 cells, despite the fact that we observed only a significant reduction of extracellular glutamate and almost any change in extracellular cystine accumulation, ERA treatment induced almost 30% of cell death and a significantly reduced ATF4 accumulation, probably justifying their greater sensitivity. The ATF4 behavior in cells treated with the drug combination is quite interesting. Indeed, since ATF4 expression may be associated with cancer cell resistance to ERA, particularly in MiaPaCa-2 cells, its decreased expression at a later time point fits well with the highest levels of cell death observed (**Figure 3E**). Its reduction may be ascribed to the prolonged UPR activation, due to FR054 treatment under stressing conditions. Indeed, CHOP-dependent apoptosis is characterized by a specific restoration of protein translation, in order to transcribe pro-apoptotic genes (54, 55). In such a condition, ATF4 becomes less active, due to either the expression of the inactive kinase *Tribbles Pseudokinase 3* (*TRIB3*), which acts as a negative feedback regulator of the ATF4-dependent transcription during the UPR (56), or its degradation by a ubiquitination-dependent mechanism (57, 58). In this regard, we would underline that FR054, as shown by the results from our RNA-seq data, is able to transcriptionally induce *TRIB3* (log_2_FoldChange: 9.53 and 1.71 in MiaPaCa-2 and BxPC3 cells, respectively), suggesting a possible *TRIB3-*dependent mechanism of ATF4’s function. Furthermore, it has been recently shown that Glycogen Synthase Kinase 3 Beta (GS3K) can target ATF4 for degradation. Such mechanism is controlled by the state of activation of AKT Serine/Threonine Kinase (AKT). Indeed, attenuation of the AKT pathway, leading to GSK3 activity, causes ATF4 degradation (59). Notably, our previous findings demonstrated that FR054 can significantly reduce AKT activation in PDAC cells (12), therefore corroborating this mechanism as a possible way to induce degradation of ATF4 and to avoid its protective role. Our findings also indicated that FR054 also reduces NRF2 expression (**Figures 4A, B**). Interestingly, GSK3 activity also controls NRF2 ubiquitination and degradation, suggesting a common mechanism of action on both NRF2 and ATF4 (60, 61). Furthermore, analysis of KEAP1 expression, a key protein in NRF2 degradation, indicated that KEAP1, in both cell lines, is significantly induced in an FR054-dependent fashion, as shown in **Figure 4A** (compare lanes 3, 4, 7, and 8 with lanes 1, 2, 5, and 6) and **Figure 4B** (compare lanes 3, 4, 7, and 8 with lanes 1, 2, 5, and 6). The parallel increase of both NRF2 and KEAP1 suggests, as previously observed, a post-induction of KEAP1 in order to turn off the NRF2 signaling (62) that ultimately fits well with the increased cell death observed in FR054-treated samples at 72 h. Regarding the observed parallel increase of NRF2 and KEAP1, it is noteworthy to observe that some authors demonstrated that persistent accumulation of NRF2 is harmful (63, 64). In this regard, some reports indicated that NRF2 is able to activate an auto-regulatory loop by inducing the expression of KEAP1 (65, 66). Intriguingly, O-linked N-acetylglucosamine (GlcNAc) transferase (OGT) activation, the only enzyme deputy to protein O-glycosylation, has been associated with NRF2-dependent stress response, since OGT inhibition may facilitate NRF2 activation (67, 68). The mechanism described for such an OGT-dependent NRF2 regulation is the O-GlcNAcylation status of KEAP1 (69). Indeed, O-GlcNAcylation of KEAP1 is required for the efficient ubiquitination and degradation of NRF2 (69). Of interest, NRF2 degradation and ferroptosis have also been recently linked through protein O-GlcNAcylation. In particular, protein O-GlcNAcylation regulates ferritinophagy and mitochondria behaviors to dictate ferroptosis; in particular, it has been shown that inhibition of O-GlcNAcylation promotes ferroptosis (70). In this scenario, we may hypothesize that ERA and FR054 cotreatment causes an overactivation of NRF2, following UPR and oxidative stress increase, as well as protein de-glycosylation (11, 12), that will eventually activate a KEAP1-dependent regulatory negative loop that causes NRF2 degradation and cellular oxidative stress. This hypothesis is supported by our observation of a robust activation of NRF2 and KEAP1 in combined treatment in both cell lines (**Figures 4A, B**, **Figures S6**, **S7**), an increased lipid peroxidation (**Figures 5C–F**), and cell death (**Figures 5A, B**). Despite the fact that we have yet to test protein expression in PANC1 cells, our hypothesis is also strongly supported in this cell line as shown in **Figure 8** in which the combined treatment induces a significant increase in cell death, inhibits SLC7A11 activity, and increases ferroptosis markers.

In cancer cells, inhibition of system SLC7A11-mediated cystine uptake by ERA may be sufficient to initiate ferroptosis by interfering with GSH and GSSG intracellular levels. Our results in the three PDAC cell lines indicated that ERA was able to induce a small but significant decrease in GSH level only in PANC1 and BxPC3 cells (**Figures 4I, J**, **8F and G**), given that, in MiaPaCa-2 cells, the decrease was unimportant. However, these findings agree with previous research, showing that PANC1 and BxPC3 cells are more sensitive to imidazole ketone erastin (IKE), an erastin analogue, as compared to MiaPaCa-2 cells, suggesting that the cell ability to avoid ERA effect is associated with GSH intracellular levels (24). Accordingly, combined treatment, able to significantly increase a ferroptosis mechanism of cell death (**Figures 5C–F**, **8H, J**), significantly reduced GSH levels in MiaPaCa-2 and PANC1 cells. Regarding the GSH level in BxPC3, it must be underlined that although we observed a significant reduction and accumulation of extracellular glutamate and cystine, respectively, in the combined treatment, we did not observe a GSH reduction, suggesting a more complex mechanism causing ferroptosis in this cell line. Of note, in all three cell lines, upon ERA and combined treatments, we observed a consistent and significant decrease of GSSG. This result is both in agreement (24, 52, 71, 72) and in disagreement with other published observations because other researchers have shown that GSH depletion is accompanied by high GSSG levels. While we do not have an explanation about our different results, we would underline that in our experimental conditions, BSO treatment also caused a significant reduction of both GSH and GSSG as compared to control cells, suggesting that the decline of total GSH may have a role in ferroptosis onset (24, 72). Whether the GSSG level plays a role in the execution of ferroptosis remains unclear. An almost completely opposite behavior was observed in FR054-treated cells. Indeed, in line with the transcriptional data and the glutamate/cystine measurements, the effect of FR054 on GSH level as compared to control cells varied among cell lines (no change in MiaPaCa-2 cells, slightly high in BxPC3 cells, and slightly low in PANC1 cells). Nevertheless, in all three cell lines upon FR054 treatment, the GSSG levels significantly increased, suggesting that these cells were actively using GSH for cellular detoxification maybe to restore protein folding and cope with UPR stress (73). In this regard, a recent manuscript has proposed an important role for GSSG in controlling endoplasmic reticulum (ER) function. Indeed, it has been shown that increased GSSG level may, directly or indirectly, alter ER oxidative protein maturation and protect ER homeostasis (74). How the level of GSSG may control ER homeostasis and ferroptosis is not the topic of this manuscript, but since few authors addressed this role, it could be the topic of a future investigation.

Nevertheless, the cell death mechanism observed in all the PDAC cell lines and particularly in the combined treatment is clearly dependent on GSH levels, since we observed that cell survival was almost completely restored or significantly reduced by adding NAC or BSO, respectively (**Figures 5A, B**). Moreover, analysis of lipid peroxidation and MDA confirmed the induction of a ferroptosis mechanism of cell death especially in the combined treatment (**Figures 5C–F**, **8H, I**). Notably, lipid peroxidation was also observed in FR054-treated cells, which was consistent with previous findings showing a crosstalk between the two processes, ER stress and ferroptosis, in controlling cell death through the activation of the PERK–eIF2α–ATF4–CHOP axis (75) or the PERK–Nrf2–HMOX1 axis (44). It was intriguing that HMOX1 was identified as one of the most regulated mRNA in both cell lines upon FR054 treatment (**Figures 1**, **2**), inferring that accumulation of ferrous iron (Fe^2+^), generated by HMOX1 activity could cause further lipid peroxidation in the combined treatment. Indeed, Western blot analysis indicated that both MiaPaCa-2 and BxPC3 cells were also induced at the protein level (**Figure 4A**). Of note, we have to highlight that although it was significantly induced at the mRNA level upon FR054 treatment in both cell lines, it was detected only in MiaPaCa-2 cells at the protein level. We do not have an explanation for this discrepancy, but we would emphasize that all the other mRNAs tested by Western blot, specifically ATF4, DDIT3/CHOP, NRF2, and SLC7A11, were modulated similarly at both the mRNA and protein levels.

The role of cellular metabolism in ferroptosis execution has been addressed in several studies as reviewed in (76), and in fact, an increasing number of metabolic pathways appear to converge on ferroptosis. Intriguingly, we show that in all the PDAC cell lines, and especially in K-Ras mutated cell lines, the combined treatment causes a metabolic rewiring leading to a reduction of glucose entry into the TCA cycle. This effect, but to a lesser extent, was observed also following the single treatments (**Figures 6A**, **7A** and **Figure S7A**). The effect correlates well with our transcriptional data in which we observed that FR054 treatment upregulates several genes of the glycolytic branches able to generate NADPH, such as *Glucose-6-Phosphate Dehydrogenase* (*G6PD*) and *6-Phosphogluconate Dehydrogenase* (*PGD*) or *Phosphoserine Aminotransferase 1 (PSAT1)* and *Phosphoglycerate Dehydrogenase (PHGDH)*, necessary for the recycling of the oxidized GSH through GSR activity in order to maintain the cellular redox homeostasis (77). Furthermore, we show that such glycolytic rewiring is associated with a concurrent increase in glutamine dependence of PDAC cells especially in K-Ras mutated MiaPaCa-2 and PANC1. The role of glutamine in ferroptosis is quite complex; however, some authors showed that glutamine drives ferroptosis in combination with cystine deprivation through glutaminolysis. Indeed, it has been proposed that the inhibition of SLC7A11 activity, which increases glutaminolysis, causes a greater glutamate conversion into α-KG that boosts the TCA cycle, the mitochondria activity, the fatty acid synthesis, and the generation of ROS that eventually altogether concur with ferroptosis (78, 79). Importantly, glutaminolysis alone is not able to trigger ferroptosis, pointing out that ferroptosis may happen only when glutaminolysis is activated in a cysteine depletion condition (79–81). In contrast, it has also been shown that ferroptosis is further accelerated by inhibition of ETC complexes and OXPHOS (71, 82). The discrepancy between previous studies and ours can be explained by the differences in cell type, ferroptosis inducers, and exposure time. In particular, activation of ferroptotic signaling in response to ferroptosis inducers (RSL3, erastin, and cysteine deprivation) might be mediated through different mechanistic pathways in mitochondria. On the other hand, other authors have shown that ferroptosis sensitivity is also associated with metabolic compartmentalization. Indeed, genetic silencing and pharmacological inhibition of glutaminolysis through GLS2, but not GLS1, or GOT1 have been shown to inhibit erastin-induced ferroptosis (79, 83, 84). Furthermore, also in our study, while the combined treatment was able to increase ferroptosis in all PDAC cells, we observed some differences between K-Ras mutated cell lines and K-Ras wild-type cells, especially regarding glutaminolysis activation (compare **Figures 6**, **7**, **Figure S7**, and **Supplementary Table 4**). A role of oncogenic K-Ras in favoring glutamine utilization has been widely described (85, 86). However, if the oncogenic K-Ras-dependent enhancement of glutamine utilization is an important cause of the increased sensitivity of MiaPaCa-2 and PANC1 cells to the combined treatment-induced ferroptosis, it needs further experiments. Indeed, as recently described, genes involved in ferroptosis also exert other functions in different cell contexts, and therefore, the role of glutamine in ferroptosis may also be dependent and not dependent on K-Ras status (87). Therefore, the differences emerging from our study and from other studies possibly reflect the incomplete understanding of how various factors dictate sensitivity to ferroptosis.

In conclusion, our findings indicate that FR054 alone or in combination with ERA is able to modulate oxidative stress response and central carbon metabolism, favoring glutaminolysis over glycolysis, which, under conditions of SLC7A11 inhibition following ERA treatment, causes imbalance in cellular redox state, GSH depletion, lipid-ROS accumulation, and finally ferroptosis. Based on our *in vitro* data, we propose that FR054, together with a drug capable of modulating the intracellular level of cysteine, such as ERA, may become a novel candidate for *in vivo* ferroptosis induction for the targeted killing of PDAC cells.

### Limitations

The aim of our study was to identify possible pathways or proteins whose inhibition/activation could synergize with FR054, a novel HBP inhibitor, in inducing PDAC cell death. However, our study contains certain limitations. First, a Q-PCR validation of some DEGs was not conducted in order to determine if some specific pathways were more activated by FR054 treatment. Second, we did not test the effect of a specific ferroptosis inhibitor such as Ferrostatin-1 to confirm the ability of the combined treatment in inducing ferroptosis. Third, we did not test our combination in immortalized pancreatic cells such as human pancreatic duct epithelial cells to support the use of the combination in preclinical *in vivo* models. Fourth, to dissect the role of glutaminolysis in the three PDAC cell lines characterized by a different K-Ras mutational status, we could test the effect of the mutated K-Ras activity reduction by using some of the available mutation-specific inhibitors (88). Finally, we did not directly assess the mitochondrial activity and the role of metabolism compartmentalization, as we have done previously (89), in our cell models with the different treatments, causing ferroptosis.

## Data availability statement

The datasets presented in this study can be found in online repositories. The names of the repository/repositories and accession number(s) can be found below: GEO database, accession number GSE223303.

## Author contributions

Conception and design: AW and FC. Development of methodology: BZ, MG, TL, AP, LB, AW, and FC. Performing research: BZ, MB, TL, VB, and AP. Analysis and interpretation of data: BZ, MG, TL, AP, LB, AW, and FC. Writing the draft of the manuscript: FC. Review of the manuscript: BZ, VB, AW, and FC. Revision of the manuscript: all authors. All authors contributed to the article and approved the submitted version.
