# Supplementary material for: PGM3 inhibition shows cooperative effects with erastin inducing pancreatic cancer cell death via activation of the unfolded protein response
Source: Front Oncol. 2023 May 16;13:1125855. doi: 10.3389/fonc.2023.1125855 (PMC10227458; doi:10.3389/fonc.2023.1125855)
Supplement: Supplementary file 1 [file DataSheet_1.pdf]

#	pathway	NES	FDR	count
1	PHOTODYNAMIC THERAPY INDUCED UNFOLDED PROTEIN RESPONSE	2.48E+00	0.00E+00	23
2	CYTOPLASMIC RIBOSOMAL PROTEINS	2.31E+00	6.57E-04	86
3	PHOTODYNAMIC THERAPY INDUCED NFE2L2 NRF2 SURVIVAL SIGNALING	2.13E+00	9.16E-03	23
4	NRF2 PATHWAY	2.11E+00	1.02E-02	104
5	TRANSCRIPTIONAL ACTIVATION BY NRF2 IN RESPONSE TO PHYTOCHEMICALS	2.03E+00	1.72E-02	13
6	EXERCISEINDUCED CIRCADIAN REGULATION	2.01E+00	1.92E-02	41
7	UNFOLDED PROTEIN RESPONSE	2.03E+00	2.04E-02	24
8	SEROTONIN AND ANXIETYRELATED EVENTS	1.98E+00	2.49E-02	6
9	OXIDATIVE STRESS RESPONSE	1.94E+00	3.45E-02	28
10	NRF2ARE REGULATION	1.93E+00	3.55E-02	21
11	OREXIN RECEPTOR PATHWAY	1.91E+00	4.01E-02	103
12	MRNA PROTEIN AND METABOLITE INDUCATION PATHWAY BY CYCLOSPORIN A	1.89E+00	4.86E-02	7
13	VITAMIN DSENSITIVE CALCIUM SIGNALING IN DEPRESSION	1.87E+00	5.61E-02	26
14	PREIMPLANTATION EMBRYO	1.83E+00	7.64E-02	37
15	FERROPTOSIS	1.82E+00	8.09E-02	59
16	GENES RELATED TO PRIMARY CILIUM DEVELOPMENT BASED ON CRISPR	-1.86E+00	8.10E-02	89
17	DEREGULATION OF RAB AND RAB EFFECTOR GENES IN BLADDER CANCER	-1.88E+00	8.90E-02	15
18	BENZOAPYRENE METABOLISM	1.80E+00	9.25E-02	7
19	MIR517 RELATIONSHIP WITH ARCN1 AND USP1	1.77E+00	1.06E-01	5
20	MRNA PROCESSING	1.76E+00	1.15E-01	124
21	TYPE I INTERFERON INDUCTION AND SIGNALING DURING SARSCOV2 INFECTION	-1.89E+00	1.27E-01	27
22	WHITE FAT CELL DIFFERENTIATION	1.73E+00	1.34E-01	29
23	DRUG INDUCTION OF BILE ACID PATHWAY	1.73E+00	1.37E-01	6
24	EUKARYOTIC TRANSCRIPTION INITIATION	1.72E+00	1.45E-01	40
25	IRON METABOLISM IN PLACENTA	1.70E+00	1.54E-01	10
26	PROTEASOME DEGRADATION	1.69E+00	1.58E-01	55
27	ANTIVIRAL AND ANTIINFLAMMATORY EFFECTS OF NRF2 ON SARSCOV2 PATHWAY	1.70E+00	1.60E-01	25
28	HYPERTROPHY MODEL	1.70E+00	1.64E-01	16
29	TRANSCRIPTIONAL CASCADE REGULATING ADIPOGENESIS	1.67E+00	1.68E-01	13
30	COHESIN COMPLEX CORNELIA DE LANGE SYNDROME	1.67E+00	1.70E-01	34
31	NEPHROTIC SYNDROME	-1.91E+00	1.75E-01	38
32	BLADDER CANCER	1.67E+00	1.76E-01	36
33	SEROTONIN AND ANXIETY	1.65E+00	1.81E-01	9
34	MIRNAS INVOLVED IN DNA DAMAGE RESPONSE	1.63E+00	1.94E-01	21
35	PARKINUBIQUITIN PROTEASOMAL SYSTEM PATHWAY	1.62E+00	1.97E-01	60
36	P53 TRANSCRIPTIONAL GENE NETWORK	1.63E+00	1.98E-01	62
37	GLUTATHIONE METABOLISM	1.63E+00	1.99E-01	15
38	CHROMOSOMAL AND MICROSATELLITE INSTABILITY IN COLORECTAL CANCER	1.64E+00	2.01E-01	70
39	HEMATOPOIETIC STEM CELL DIFFERENTIATION	1.61E+00	2.01E-01	42
40	NUCLEAR RECEPTORS METAPATHWAY	1.63E+00	2.03E-01	234
41	GANGLIO SPHINGOLIPID METABOLISM	1.58E+00	2.41E-01	9
42	STRIATED MUSCLE CONTRACTION PATHWAY	1.58E+00	2.44E-01	24
43	LET7 INHIBITION OF ES CELL REPROGRAMMING	1.58E+00	2.50E-01	8

Figure S1. Table listing all significative enriched gene sets in treated MIAPaCa-2 cells. Rankings based on FDR score. ± NES indicates upregulation or downregulation respectively of gene set in treated MIAPaCa-2 cells.

Figure S1
