# Supplementary material for: PGM3 inhibition shows cooperative effects with erastin inducing pancreatic cancer cell death via activation of the unfolded protein response
Source: Front Oncol. 2023 May 16;13:1125855. doi: 10.3389/fonc.2023.1125855 (PMC10227458; doi:10.3389/fonc.2023.1125855)
Supplement: Supplementary file 2 [file DataSheet_2.pdf]

#	pathway	enrichment	pvalue	count
1	FERROPTOSIS	2.53E+00	0.00E+00	60
2	OVERVIEW OF PROINFLAMMATORY AND PROFIBROTIC MEDIATORS	-1.99E+00	0.00E+00	66
3	NETWORK MAP OF SARSCOV2 SIGNALING PATHWAY	-2.06E+00	0.00E+00	166
4	SARSCOV2 INNATE IMMUNITY EVASION AND CELLSPECIFIC IMMUNE RESPONSE	-2.13E+00	0.00E+00	55
5	ALLOGRAFT REJECTION	-2.26E+00	0.00E+00	48
6	PHOTODYNAMIC THERAPYINDUCED NFE2L2 NRF2 SURVIVAL SIGNALING	2.30E+00	1.00E-03	23
7	TYPE II INTERFERON SIGNALING IFNG	-1.98E+00	1.00E-03	29
8	CYTOKINES AND INFLAMMATORY RESPONSE	-1.91E+00	3.00E-03	17
9	IMMUNE RESPONSE TO TUBERCULOSIS	-1.92E+00	3.00E-03	23
10	TRANSCRIPTIONAL ACTIVATION BY NRF2 IN RESPONSE TO PHYTOCHEMICALS	2.13E+00	4.00E-03	14
11	NRF2ARE REGULATION	2.10E+00	4.00E-03	22
12	BENZOAPYRENE METABOLISM	2.10E+00	4.00E-03	8
13	ESTROGEN RECEPTOR PATHWAY	2.07E+00	5.00E-03	11
14	HIPPOMERLIN SIGNALING DYSREGULATION	-1.85E+00	9.00E-03	94
15	SPINAL CORD INJURY	-1.86E+00	9.00E-03	89
16	MIRNAS INVOLVED IN DNA DAMAGE RESPONSE	2.02E+00	1.00E-02	23
17	CHEMOKINE SIGNALING PATHWAY	-1.81E+00	1.50E-02	128
18	MBDNF AND PROBDNF REGULATION OF GABA NEUROTRANSMISSION	-1.82E+00	1.50E-02	28
19	INFLAMMATORY RESPONSE PATHWAY	-1.80E+00	1.60E-02	21
20	CCL18 SIGNALING PATHWAY	-1.81E+00	1.60E-02	39
21	AUTOPHAGY	1.95E+00	2.00E-02	29
22	MIR5093P ALTERATION OF YAP1ECM AXIS	-1.78E+00	2.00E-02	14
23	DEVELOPMENT OF URETERIC COLLECTION SYSTEM	-1.77E+00	2.40E-02	50
24	OXIDATIVE STRESS RESPONSE	1.91E+00	3.00E-02	29
25	NEURODEGENERATION WITH BRAIN IRON ACCUMULATION NBIA SUBTYPES PATHWAY	1.90E+00	3.10E-02	44
26	NICOTINE EFFECT ON DOPAMINERGIC NEURONS	-1.72E+00	4.00E-02	14
27	COMPLEMENT SYSTEM	-1.73E+00	4.00E-02	58
28	COVID19 ADVERSE OUTCOME PATHWAY	-1.73E+00	4.00E-02	9
29	TYPE I INTERFERON INDUCTION AND SIGNALING DURING SARSCOV2 INFECTION	-1.74E+00	4.00E-02	26
30	OLIGODENDROCYTE SPECIFICATION AND DIFFERENTIATION LEADING TO MYELIN COMPONENTS FOR CNS	-1.72E+00	4.20E-02	18
31	PATHOGENESIS OF SARSCOV2 MEDIATED BY NSP9NSP10 COMPLEX	-1.73E+00	4.20E-02	14
32	COMPLEMENT AND COAGULATION CASCADES	-1.71E+00	4.30E-02	40
33	COMPLEMENT SYSTEM IN NEURONAL DEVELOPMENT AND PLASTICITY	-1.71E+00	4.40E-02	79
34	SELECTIVE EXPRESSION OF CHEMOKINE RECEPTORS DURING TCELL POLARIZATION	-1.70E+00	5.10E-02	15
35	PRIMARY FOCAL SEGMENTAL GLOMERULOSCLEROSIS FSGS	-1.69E+00	5.60E-02	65
36	EBOLA VIRUS INFECTION IN HOST	-1.68E+00	6.10E-02	108
37	GLUTATHIONE METABOLISM	1.82E+00	6.20E-02	16
38	GPCRS CLASS A RHODOPSINLIKE	-1.66E+00	6.80E-02	84
39	NONGENOMIC ACTIONS OF 125 DIHYDROXYVITAMIN D3	-1.66E+00	6.80E-02	62
40	BURN WOUND HEALING	-1.66E+00	7.00E-02	82
41	HIPPO SIGNALING REGULATION PATHWAYS	-1.65E+00	7.30E-02	79
42	NEOVASCULARISATION PROCESSES	-1.65E+00	7.40E-02	34
43	PLATELETMEDIATED INTERACTIONS WITH VASCULAR AND CIRCULATING CELLS	-1.64E+00	8.30E-02	12
44	WNT SIGNALING	-1.63E+00	8.40E-02	96
45	MIRNA TARGETS IN ECM AND MEMBRANE RECEPTORS	-1.63E+00	8.50E-02	24
46	CARDIAC PROGENITOR DIFFERENTIATION	-1.63E+00	8.50E-02	34
47	FOCAL ADHESION	-1.63E+00	8.60E-02	170

Figure S2

#	pathway	enrichment	pvalue	count
48	LUNG FIBROSIS	-1.62E+00	9.10E-02	40
49	AIRWAY SMOOTH MUSCLE CELL CONTRACTION	-1.62E+00	9.20E-02	15
50	NRF2 PATHWAY	1.78E+00	9.30E-02	110
51	ARYL HYDROCARBON RECEPTOR PATHWAY WP2873	1.77E+00	9.60E-02	38
52	EDA SIGNALING IN HAIR FOLLICLE DEVELOPMENT	-1.61E+00	1.01E-01	12
53	HAIR FOLLICLE DEVELOPMENT CYTODIFFERENTIATION PART 3 OF 3	-1.60E+00	1.01E-01	64
54	NEPHROTIC SYNDROME	-1.60E+00	1.02E-01	36
55	BMP2WNT4FOXO1 PATHWAY IN PRIMARY ENDOMETRIAL STROMAL CELL DIFFERENTIATION	-1.60E+00	1.03E-01	11
56	SARS CORONAVIRUS AND INNATE IMMUNITY	-1.59E+00	1.18E-01	17
57	VITAMIN B12 METABOLISM	-1.58E+00	1.20E-01	37
58	HEDGEHOG SIGNALING PATHWAY WP4249	-1.58E+00	1.23E-01	39
59	ESTROGEN METABOLISM	1.72E+00	1.32E-01	14
60	PROSTAGLANDIN SIGNALING	-1.56E+00	1.36E-01	24
61	GENES TARGETED BY MIRNAS IN ADIPOCYTES	-1.57E+00	1.37E-01	10
62	TGFBETA SIGNALING IN THYROID CELLS FOR EPITHELIALMESENCHYMAL TRANSITION	-1.56E+00	1.39E-01	16
63	LNCRNA IN CANONICAL WNT SIGNALING AND COLORECTAL CANCER	-1.56E+00	1.39E-01	81
64	PLURIPOTENT STEM CELL DIFFERENTIATION PATHWAY	-1.56E+00	1.42E-01	34
65	PATHWAYS OF NUCLEIC ACID METABOLISM AND INNATE IMMUNE SENSING	-1.55E+00	1.42E-01	12
66	ARRHYTHMOGENIC RIGHT VENTRICULAR CARDIOMYOPATHY	-1.55E+00	1.43E-01	54
67	MAMMALIAN DISORDER OF SEXUAL DEVELOPMENT	-1.55E+00	1.48E-01	17
68	VITAMIN B12 DISORDERS	-1.54E+00	1.58E-01	11
69	NEURAL CREST CELL MIGRATION IN CANCER	-1.54E+00	1.59E-01	36
70	HOSTPATHOGEN INTERACTION OF HUMAN CORONAVIRUSES INTERFERON INDUCTION	-1.53E+00	1.61E-01	32
71	IL1 AND MEGAKARYOCYTES IN OBESITY	-1.53E+00	1.65E-01	20
72	WNT SIGNALING IN KIDNEY DISEASE	-1.52E+00	1.80E-01	31
73	GENES ASSOCIATED WITH THE DEVELOPMENT OF RHEUMATOID ARTHRITIS	-1.52E+00	1.81E-01	11
74	PHOTODYNAMIC THERAPYINDUCED UNFOLDED PROTEIN RESPONSE	1.67E+00	1.89E-01	25
75	NCRNAS INVOLVED IN WNT SIGNALING IN HEPATOCELLULAR CARCINOMA	-1.50E+00	2.07E-01	74
76	CELLS AND MOLECULES INVOLVED IN LOCAL ACUTE INFLAMMATORY RESPONSE	-1.50E+00	2.08E-01	12
77	PI3K AKT mTOR SIGNALING PATHWAY AND THERAPEUTIC OPPORTUNITIES	1.64E+00	2.12E-01	29
78	ZINC HOMEOSTASIS	-1.50E+00	2.12E-01	29
79	FIBRIN COMPLEMENT RECEPTOR 3 SIGNALING PATHWAY	-1.50E+00	2.14E-01	33
80	ANGIOTENSIN II RECEPTOR TYPE 1 PATHWAY	-1.49E+00	2.24E-01	23
81	MALIGNANT PLEURAL MESOTHELIOMA	-1.48E+00	2.28E-01	371
82	TRANSCRIPTION COFACTORS SKI AND SKIL PROTEIN PARTNERS	1.60E+00	2.32E-01	18
83	AFLATOXIN B1 METABOLISM	1.61E+00	2.34E-01	6
84	METAPATHWAY BIOTRANSFORMATION PHASE I AND II	1.56E+00	2.35E-01	115
85	CYSTEINE AND METHIONINE CATABOLISM	1.60E+00	2.40E-01	11
86	ARYL HYDROCARBON RECEPTOR PATHWAY WP2586	1.56E+00	2.41E-01	42
87	CYTOSOLIC DNASENSING PATHWAY	-1.47E+00	2.41E-01	52
88	CANNABINOID RECEPTOR SIGNALING	1.61E+00	2.42E-01	24
89	ANTIVIRAL AND ANTIINFLAMMATORY EFFECTS OF NRF2 ON SARSCOV2 PATHWAY	1.55E+00	2.44E-01	26
90	OSTEOBLAST SIGNALING	-1.47E+00	2.45E-01	7
91	PILOCYTIC ASTROCYTOMA	1.56E+00	2.46E-01	6
92	mRNA PROTEIN AND METABOLITE INDUCATION PATHWAY BY CYCLOSPORIN A	1.61E+00	2.48E-01	7
93	DISORDERS OF FOLATE METABOLISM AND TRANSPORT	-1.47E+00	2.48E-01	12
94	SARSCOV2 REPLICATION ORGANELLE FORMATION	1.57E+00	2.49E-01	6
95	GLYCEROPHOSPHOLIPID BIOSYNTHETIC PATHWAY	1.54E+00	2.49E-01	27

Figure S2. Table listing all significative enriched gene sets in treated BxPC3 cells. Rankings based on FDR score. ± NES indicates upregulation or downregulation respectively of gene set in treated BxPC3 cells.

Figure S2
