# Supplementary figures and images for: PGM3 inhibition shows cooperative effects with erastin inducing pancreatic cancer cell death via activation of the unfolded protein response
Source: Front Oncol. 2023 May 16;13:1125855. doi: 10.3389/fonc.2023.1125855 (PMC10227458; doi:10.3389/fonc.2023.1125855)

**A**

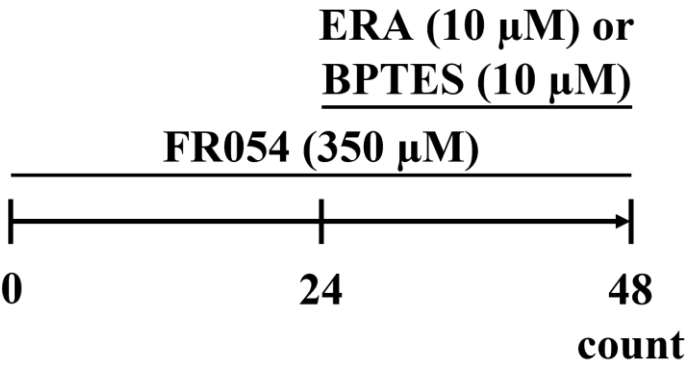

**B**

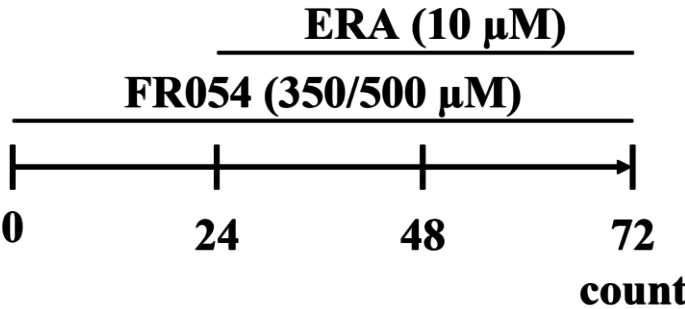

**C**

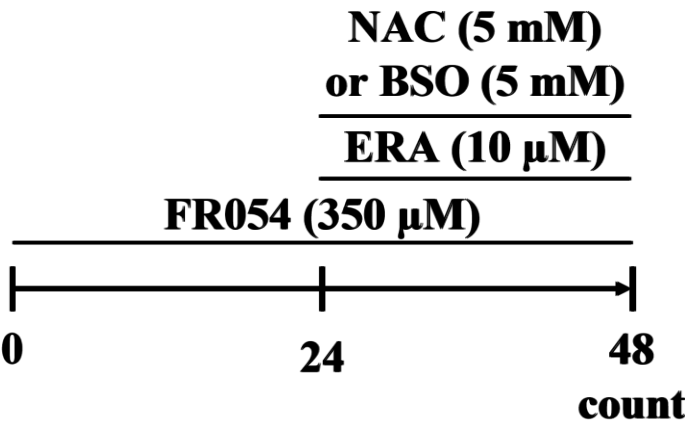

**D**

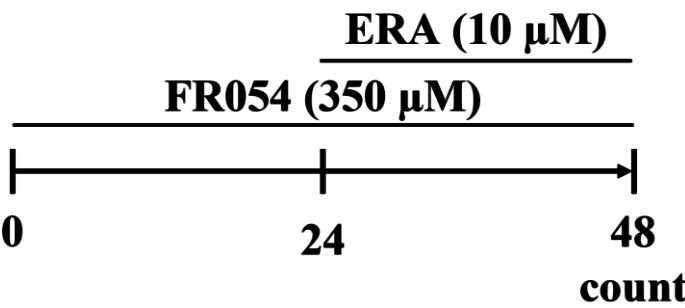

**Figure S3.** Experimental setting of the different treatments described in the main text.

Supplement: Supplementary file 3 [file DataSheet_3.pdf]
