# Supplementary material for: PGM3 inhibition shows cooperative effects with erastin inducing pancreatic cancer cell death via activation of the unfolded protein response
Source: Front Oncol. 2023 May 16;13:1125855. doi: 10.3389/fonc.2023.1125855 (PMC10227458; doi:10.3389/fonc.2023.1125855)
Supplement: Supplementary file 4 [file DataSheet_4.pdf]

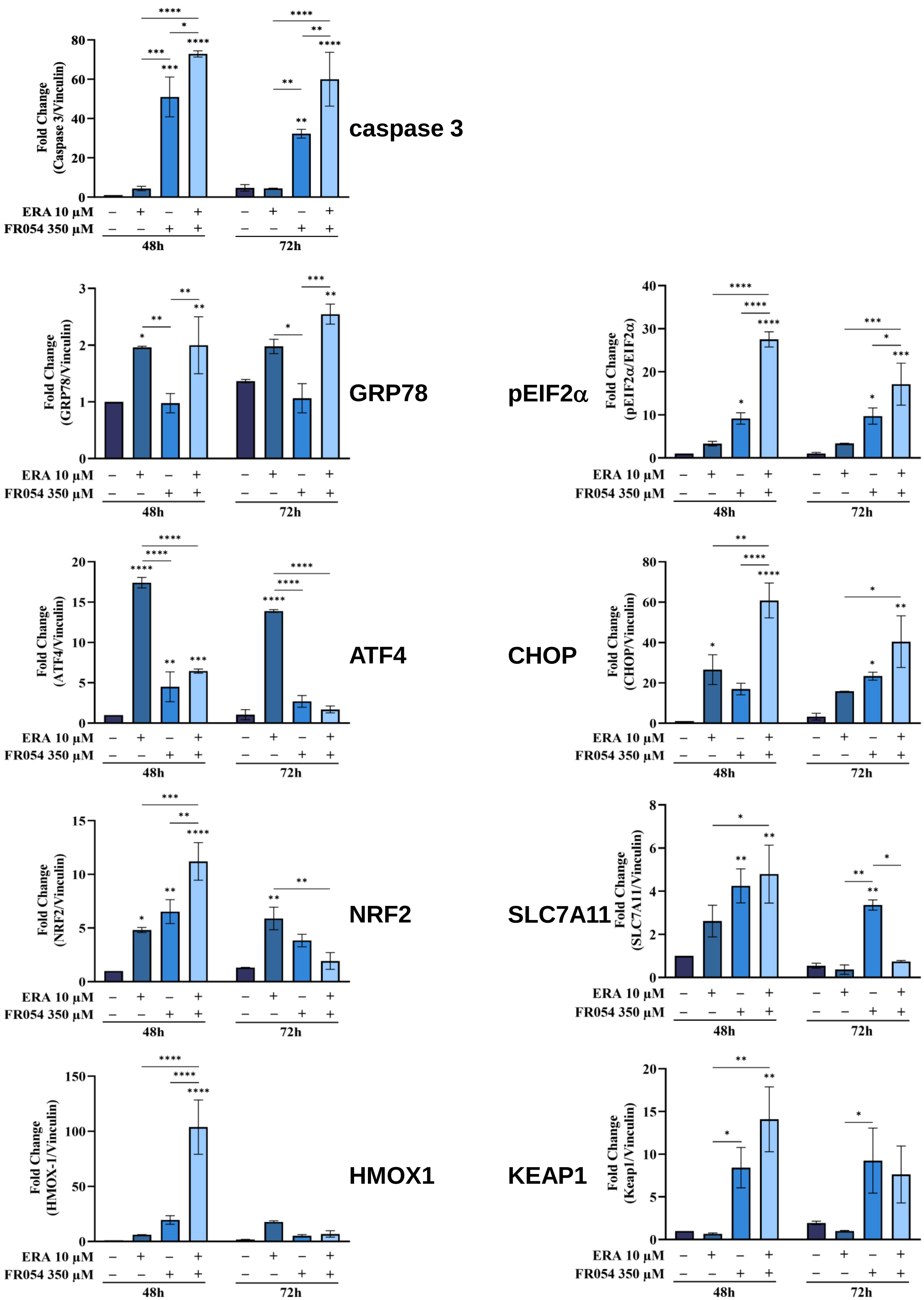
caspase 3
Fold Change (Caspase 3/Vinculin)
GRP78
Fold Change (GRP78/Vinculin)
pEIF2α
Fold Change (pEIF2α/EIF2α)
ATF4
Fold Change (ATF4/Vinculin)
CHOP
Fold Change (CHOP/Vinculin)
NRF2
Fold Change (NRF2/Vinculin)
SLC7A11
Fold Change (SLC7A11/Vinculin)
HMOX1
Fold Change (HMOX-1/Vinculin)
KEAP1
Fold Change (Keap1/Vinculin)
ERA 10 μM
FR054 350 μM
48h
72h

Figure S4

Figure S4. The combined treatment of MiaPaca-2 cells with FR054 and erastin affects UPR and oxidative stress response proteins expression. Densitometric analysis of immunoblot showing the effects of the two compounds on different UPR and oxidative stress response proteins evaluated at 48h and 72h post ERA, FR054 and their combination treatments. The immunoblot images are quantified by ImageJ software. The data are presented as mean ± SD from three independent experiments. The statistical significance was performed with two-way ANOVA with Tukey's multiple comparisons test. *$p < 0.05$, **$p < 0.01$ ***$p < 0.001$, ****$p < 0.0001$.

Figure S4
