# Supplementary material for: PGM3 inhibition shows cooperative effects with erastin inducing pancreatic cancer cell death via activation of the unfolded protein response
Source: Front Oncol. 2023 May 16;13:1125855. doi: 10.3389/fonc.2023.1125855 (PMC10227458; doi:10.3389/fonc.2023.1125855)
Supplement: Supplementary file 6 [file DataSheet_6.pdf]

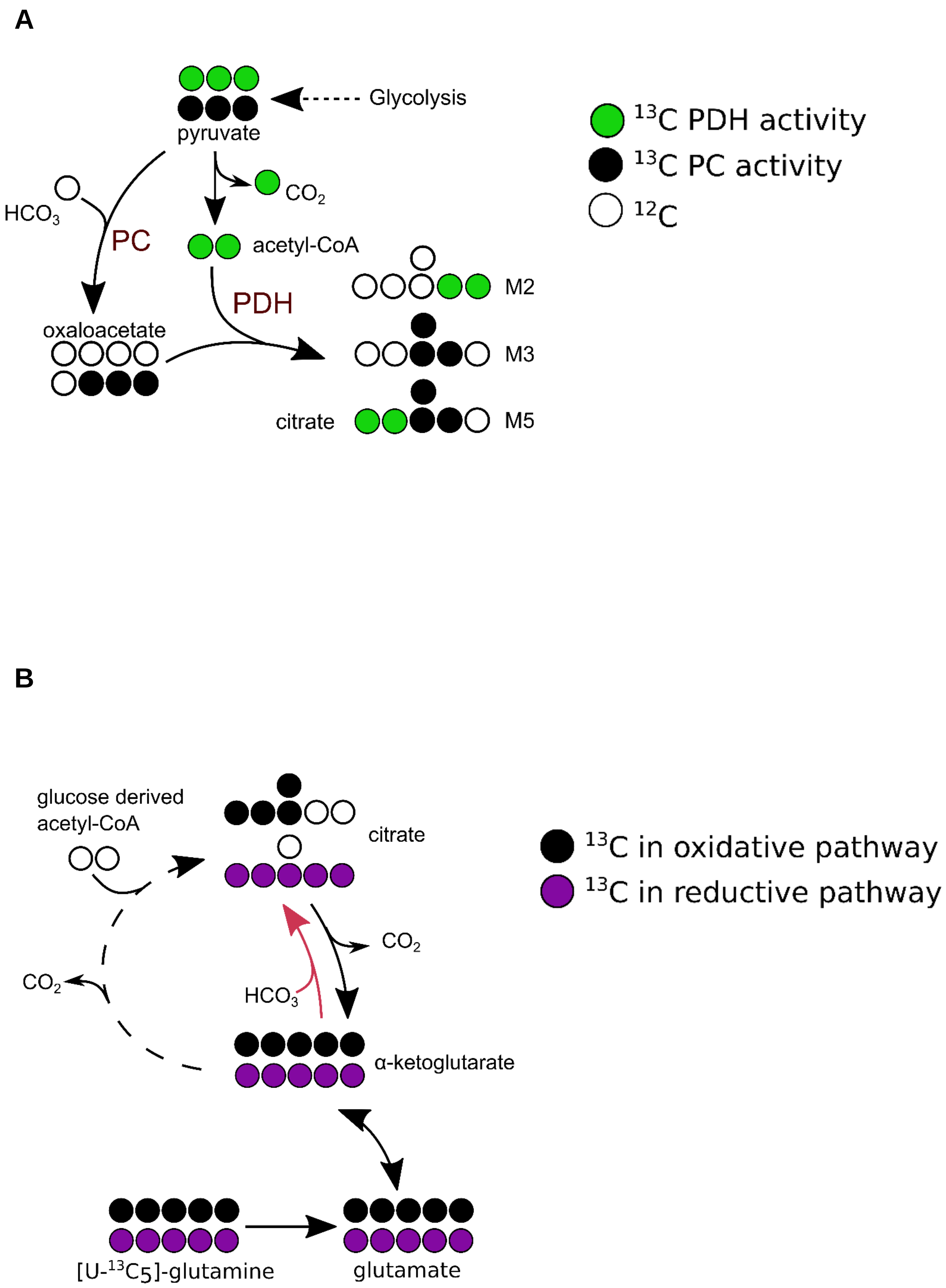

Figure S6. Isotopomers labeling for metabolomics study in MiaPaCa-2, BxPC3 and PANC1 cell lines. (A) Picture describing pyruvate, citrate, α-ketoglutarate and glutamate isotopomers from [U-13]-glucose or (B) [U-13]-glutamine. For more information refer to main text.

Figure S6
