# Supplementary material for: PGM3 inhibition shows cooperative effects with erastin inducing pancreatic cancer cell death via activation of the unfolded protein response
Source: Front Oncol. 2023 May 16;13:1125855. doi: 10.3389/fonc.2023.1125855 (PMC10227458; doi:10.3389/fonc.2023.1125855)
Supplement: Supplementary file 7 [file DataSheet_7.pdf]

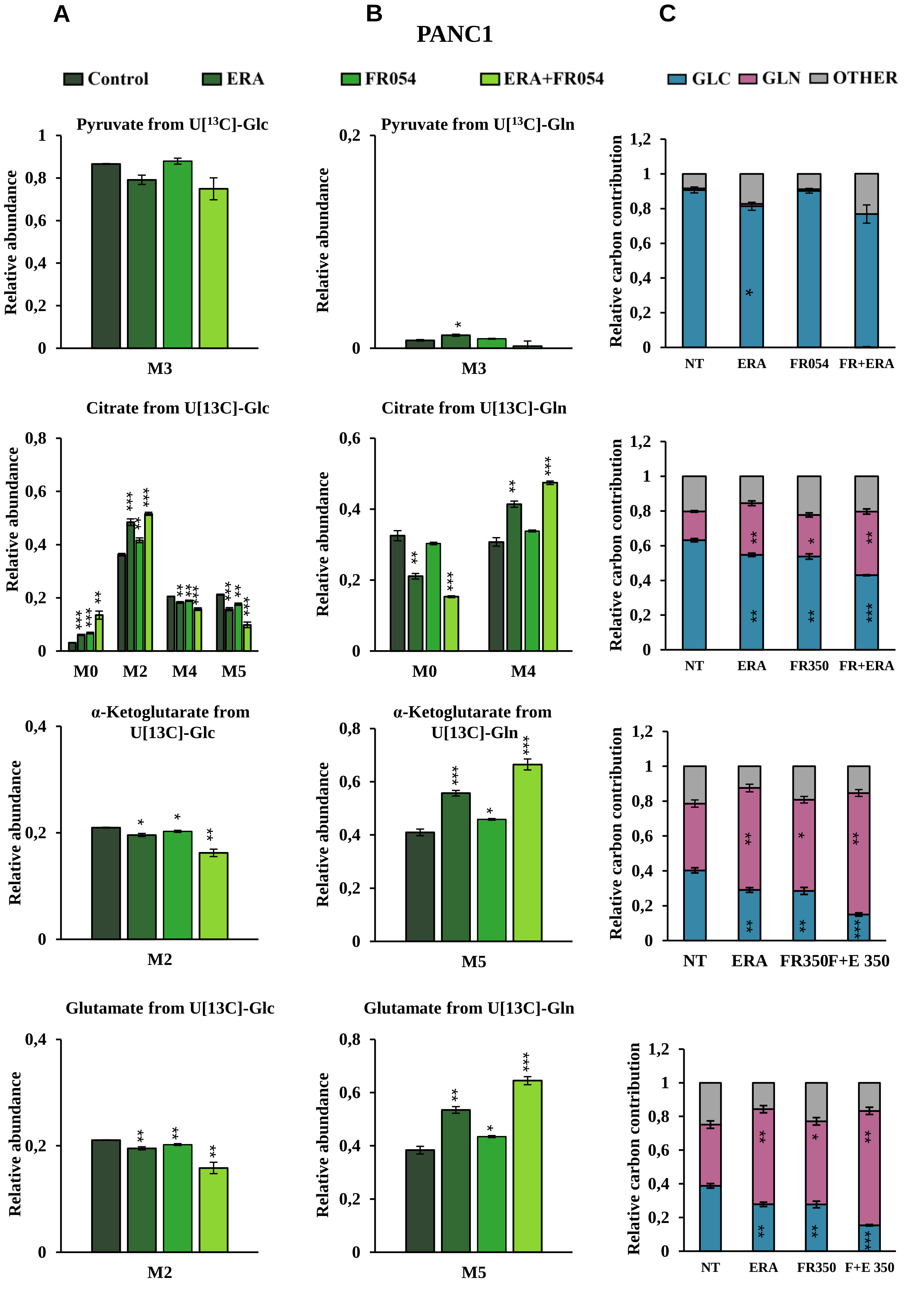
A
B
C
PANC1
Control
ERA
FR054
ERA+FR054
GLC
GLN
OTHER
Pyruvate from U[13C]-Glc
Pyruvate from U[13C]-Gln
Citrate from U[13C]-Glc
Citrate from U[13C]-Gln
α-Ketoglutarate from U[13C]-Glc
α-Ketoglutarate from U[13C]-Gln
Glutamate from U[13C]-Glc
Glutamate from U[13C]-Gln
Relative abundance
Relative carbon contribution
M0
M2
M3
M4
M5
NT
ERA
FR054
FR+ERA
FR350
F+E 350

Figure S7

Figure S7. Combined treatment in PANC1 cells favors glutaminolysis over glycolysis. (A) and (B) ^{13}C labeling of pyruvate, citrate, a-ketoglutarate and glutamate from PANC1 cells incubated in [U-$^{13}C_6$]-glucose or [U-^{13}C5]-glutamine medium for 48h. (C) Relative carbon fractional contribution of glucose and glutamine to pyruvate, citrate, a-ketoglutarate and glutamate formation. The data are presented as mean ± SEM from three independent experiments. The statistical significance was performed with Students T-Test. *$p < 0.05$, **$p < 0.01$ ***$p < 0.001$.

Figure S7
